# Eukaryotic cell-free protein synthesis: Chassis diversification, system engineering, and emerging applications

**DOI:** 10.1016/j.synbio.2026.04.022

**Published:** 2026-05-21

**Authors:** Shengxin Tong, Yiming Wang, Xin Wang, Xiaowen Jin, Duoduo Tan, Ze Wang, Yuan Lu

**Affiliations:** a College of Life Sciences, Shenyang Normal University, Shenyang, 110034, Liaoning, China; b Department of Chemical Engineering, Tsinghua University, Beijing, 100084, China; c State Key Laboratory of Green Biomanufacturing, Department of Chemical Engineering, Tsinghua University, Beijing, 100084, China; d Key Laboratory of Industrial Biocatalysis, Ministry of Education, Department of Chemical Engineering, Tsinghua University, Beijing, 100084, China

**Keywords:** Cell-free synthetic biology, Eukaryotic cell-free protein synthesis, Chassis engineering, Protein engineering, Material integration, Biomanufacturing

## Abstract

The eukaryotic cell-free protein synthesis (CFPS) system, endowed with intrinsic post-translational modification capabilities and a complex molecular chaperone network, efficiently synthesizes functional proteins with correct conformation and biological activity. This effectively compensates for the structural limitations of prokaryotic systems in expressing complex eukaryotic proteins. This paper aims to comprehensively review and analyze the latest advances in the field of eukaryotic CFPS from a systems engineering perspective. The paper delves into the diversification of host chassis, rational design of core reaction components, and the pivotal role of novel biomaterial integration and high-throughput reaction equipment development in system reconfiguration. At the application level, it summarizes the platform's latest achievements, including elucidation of fundamental mechanisms, complex protein engineering, and metabolic synthesis. It particularly highlights its potential in emerging areas such as the construction of artificial cells, the development of bioelectronic interfaces, and the design of microarray chips. Furthermore, addressing the current standardization and cost bottlenecks hindering industrialization, this paper proposes a solution strategy based on artificial intelligence and synthetic biology tools, aligning with the shift from empirical trial-and-error to rational design paradigms. By integrating the current technological landscape with emerging trends, this review aims to provide theoretical references and practical guidance for constructing an economical, high-throughput eukaryotic cell-free biomanufacturing platform.

## Highlights

• Eukaryotic cell-free protein synthesis (CFPS) plays a versatile role in synthetic biology.
• The latest advances of eukaryotic CFPS are reviewed from a systems engineering perspective.
• It investigates host chassis diversification, core component design, and material integration.
• Emerging potentials include artificial cells, bioelectronic interfaces, and microarray chips.
• Engineering strategies and paradigms are proposed to address issues in basic and applied fields.

## Graphical abstract

**Figure:**
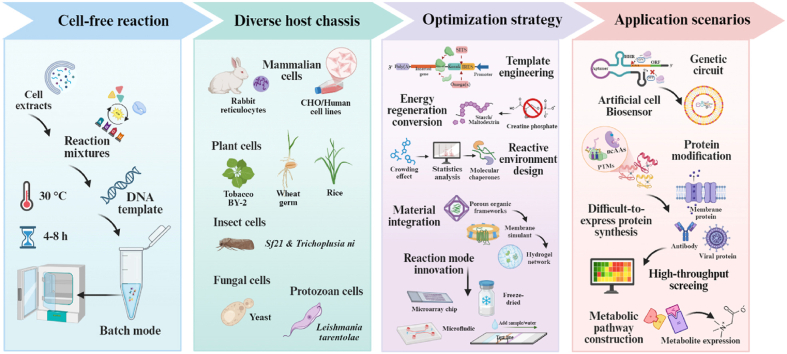

## 1 Introduction

Cell-free protein synthesis (CFPS), as a core technology in synthetic biology, offers numerous advantages, including openness, high efficiency, flexibility, and controllability. It holds extensive applications in metabolic engineering, minimal cell assembly, recombinant protein production, and the development of *in vitro* diagnostic biosensors [1]. Compared to traditional cellular systems, CFPS systems possess four major advantages: safety, tolerance, storability, and rapid response. First, CFPS systems avoid biosafety concerns by eliminating the need for live, genetically modified cells. Second, they can operate in the presence of toxins, demonstrating high tolerance to various chemical or biological agents. Additionally, CFPS systems enable long-term stable storage through freeze-drying technology. Finally, the open-environment characteristic of CFPS breaks down barriers to substance transport across membranes, facilitating rapid responses between target substances and reaction systems [2]. Leveraging these advantages, CFPS technology has become an effective means to accelerate the advancement of synthetic biology.

Modern CFPS platforms have evolved into two distinct systems: crude cell extract systems and purified recombinant protein expression (PURE) systems. Crude cell extract systems are categorized by host chassis into prokaryotic and eukaryotic sources [3]. Prokaryotic cell-free systems offer advantages such as low cost, simple preparation, high protein expression efficiency, and technical maturity. *Escherichia coli* cell-free extracts are particularly popular due to their affordability and scalability [4]. However, their low oxidative folding efficiency, limited molecular chaperone function, and absence of eukaryotic post-translational modifications (PTMs) pose challenges for folding complex proteins in prokaryotic CFPS systems [5]. Fortunately, eukaryotic cell-free systems support complex PTM processes and have been shown to accelerate the generation of functional proteins. When integrated with endogenous microsomal structures, these systems enable transmembrane protein transport, making them effective platforms for expressing multi-domain complex proteins [6]. Although the preparation of eukaryotic extracts is relatively complex and protein synthesis efficiency remains lower than that of prokaryotic CFPS systems, continuous performance optimization has driven their adaptation for high-throughput protein expression and large-scale production in complex environments. Unlike crude cell extracts, the PURE system is a fully reconstituted, highly streamlined CFPS platform. Rather than starting from cell extracts, it is reconstituted from purified individual components sourced from *E. coli* or other origins. This system offers greater precision in component composition, superior stability, safety, and flexibility, making it more advantageous for fundamental biological research [2]. Due to the complex and costly purification process of components in the PURE system, crude cell extract systems remain the most widely used CFPS platform.

Eukaryotic CFPS systems have emerged as a highly promising platform in synthetic biology due to their unique advantages of combining eukaryotic translation capabilities with an open reaction environment. In response to the growing demand for complex functional proteins in biomedical and industrial fields, researchers have expanded diverse eukaryotic chassis—from mammalian cells, fungi, plants, and insects to protozoa—developing customized systems that offer both cost-effectiveness and high fidelity [[7], [8], [9], [10], [11]]. After half a century of optimization, this field has achieved remarkable progress in system construction and reaction mode innovation. On one hand, through rational design of genetic templates, optimization of energy regeneration systems, and precise regulation of reaction environments, system efficiency and stability have been significantly enhanced [9,[12], [13], [14], [15], [16], [17], [18], [19]]. On the other hand, the introduction of advanced materials such as porous framework materials, hydrogels, and membrane mimics has not only provided protective microenvironments and functionalized interfaces for enzymes and membrane proteins but also driven the evolution of reaction modes from microbatch to high-throughput microfluidics and portable freeze-drying approaches [10,[20], [21], [22], [23], [24], [25], [26], [27], [28], [29], [30]]. With advancements in reactor technology and the development of automated equipment, the eukaryotic CFPS system has successfully expanded its application boundaries into cutting-edge fields such as genetic circuits, biomanufacturing, artificial sensing, and metabolic engineering [[31], [32], [33], [34], [35], [36], [37]].

Despite significant progress in optimizing eukaryotic CFPS systems over the past decades, their transition from laboratory tools to universal biomanufacturing platforms remains inadequate. This is primarily due to constraints across multiple dimensions, including economic viability, biological fidelity, operational scalability, and mechanistic understanding. Specifically, the field is currently constrained by the trade-off between yield and cost, difficulties in replicating complex synthetic pathways, the lack of standardization required for automation, and limitations in reaction control due to insufficient understanding of mechanisms [19,31]. To overcome these obstacles, the development of eukaryotic CFPS systems should shift from optimizing individual components toward rational engineering design. By integrating genome editing, artificial intelligence algorithms, and advanced materials science, the creation of highly biocompatible synthetic scaffolds and integrated reaction apparatus will progressively achieve the convergence of economic efficiency and high performance in eukaryotic CFPS systems [38,39]. This will enable high-throughput automated applications in complex environments. As performance and functionality deepen, the application potential of eukaryotic CFPS systems expands across broader dimensions. Beyond advancing our understanding of multigenic cascade regulation, these systems demonstrate significant translational value toward industrial applications in frontier fields such as complex protein engineering, metabolic pathway assembly, and biosensing and theranostics.

**Fig. 1:**
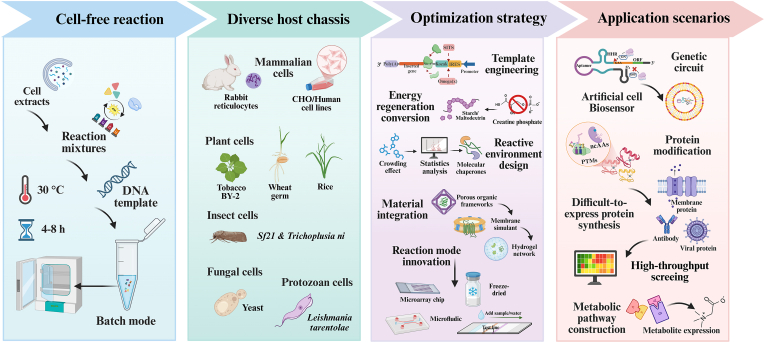
Schematic diagram of eukaryotic CFPS. The eukaryotic CFPS system meets the growing demand for complex functional proteins in biomedical and industrial fields by combining the advantages of a rapidly responsive open environment with sophisticated eukaryotic translation capabilities. The platform has expanded its diverse extract chassis from mammals and plants to insects, fungi, and protozoa. Through rational design of gene templates, energy regeneration systems, and reaction environments, performance continues to improve. Combining novel materials with innovative reaction modes has driven the expansion of its application scenarios, demonstrating value in artificial biosensing, biomanufacturing, high-throughput screening, and metabolic pathway assembly. Created with BioRender.com.

Unlike previous reviews that focused on single or fundamental eukaryotic platforms, this paper aims to provide a more comprehensive and systematic discussion, with particular emphasis on the engineering evolution and interdisciplinary technological integration of eukaryotic CFPS systems (Fig. 1). Building upon a systematic review of its nearly 70-year development history and the characteristics of diverse host chassis, the paper emphasizes key drivers such as rational design of core system components, integration of advanced materials, and reactor equipment upgrades. It also delves into the latest advances in eukaryotic CFPS applications across fundamental life science analysis, complex protein engineering, and metabolic engineering, while analyzing systemic bottlenecks hindering its transition from laboratory technology to a universal production platform. By integrating the shift from empirical optimization to rational design paradigms, particularly through emerging tools like synthetic biology and artificial intelligence, this paper aims to provide insights for addressing critical challenges such as cost, fidelity, and standardization. This will accelerate the deep application of eukaryotic CFPS technology in biomanufacturing and biopharmaceutical fields.

## 2 Construction of eukaryotic CFPS systems

### 2.1 Primary components

**Fig. 2:**
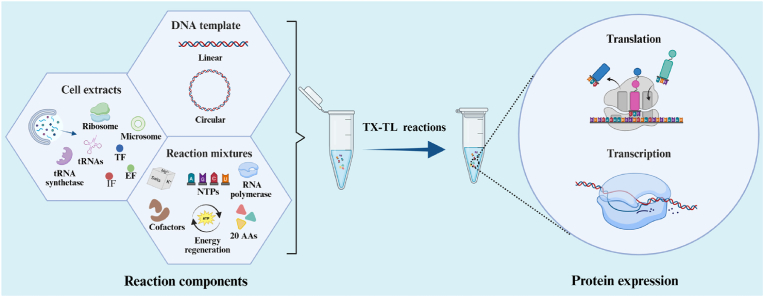
Components of the eukaryotic CFPS system and the transcription-translation process. Based on cell extracts, a reaction mixture comprising an energy regeneration system, NTPs, amino acids, RNA polymerase, and cofactors was supplemented to construct a CFPS system in microcentrifuge tubes, thereby completing the TX-TL process of the template DNA. Created with BioRender.com.

The main components of the eukaryotic CFPS system include eukaryotic cell extracts with translational activity, exogenous DNA templates, and other reaction mixtures. As the core of the eukaryotic CFPS system, cell extracts, which undergo multi-step separation to remove insoluble substances such as cellular debris, provide the necessary transcriptional and translational machinery for *in vitro* protein synthesis. This includes ribosomes, translational factors, tRNAs, and aminoacyl-tRNA synthetases. Certain eukaryotic extracts also contain endogenous vesicular structures that support protein translocations. Additionally, other supplementary reaction components are required to sustain the eukaryotic CFPS system, including HEPES-KOH buffer, energy regeneration system, 20 amino acids (AAs), exogenous RNA polymerase, nucleotide triphosphate mixtures (NTPs), dithiothreitol, salts such as K^+^ and Mg^2+^, and other cofactors. Finally, linear or circular DNA templates encoding the target protein are added to the assembled reaction components to initiate the standard CFPS reaction at 30 °C [40]. Beyond essential reaction components, various eukaryotic CFPS systems require maintaining redox conditions and supplementing with effectors, such as molecular chaperones or serine protease inhibitors, to enhance *in vitro* protein translation efficiency. The components of the eukaryotic CFPS system and the transcription-translation (TX-TL) process are shown in Fig. 2. In addition to the laboratory version of the eukaryotic CFPS systems, an increasing number of commercial eukaryotic CFPS have emerged, which play a significant role in the field of protein engineering [31].

### 2.2 System advantage

Due to significant differences in host platforms, eukaryotic and prokaryotic cell-free systems exhibit distinct focuses within the CFPS framework. Functional differences between prokaryotic and eukaryotic cell-free systems are summarized in Table 1. As shown, the primary advantages of prokaryotic cell-free systems include: rapid high-yield synthesis of target proteins, low cost, simple templates, and mature, stable technology. In contrast, eukaryotic cell-free systems retain native microsomal vesicle architecture, enabling simulation of the endoplasmic reticulum environment [18,41,42]. This facilitates critical modifications such as N-linked glycosylation, signal peptide cleavage, and correct disulfide bond formation, resulting in eukaryotic proteins with full biological activity [9,43]. The preserved microsomal structure supports the translocation of proteins bearing specific signal peptides into the lumen during translation, providing a platform for the functional characterization of membrane proteins [44,45]. Second, eukaryotic cell-free systems exhibit superior protein folding capabilities and extremely low endogenous mRNA levels, significantly enhancing the probability of expressing soluble, functionally active proteins [46]. Additionally, the codon usage patterns of eukaryotic cell-free systems more closely resemble those of mammalian cells, ensuring superior compatibility with eukaryotic genes. Compared to prokaryotic systems, their lower levels of endogenous contaminants eliminate the risk of endotoxin contamination.

**Table 1:** Comparison of eukaryotic CFPS system and prokaryotic CFPS system.

Comparison items	Prokaryotic	Eukaryotic	References
Protein yield and efficiency	Relatively high, with a yield exceeding 1 mg/mL	Relatively low, with yields typically ranging from 50 to 300 μg/mL	[18,43,52,53]
Cost and scalability	Suitable for large-scale, low-cost production and application; the single reaction using a commercial prokaryotic CFPS assay kit costs $1.7	Culturing eukaryotic cells or tissues is costly and involves a complex extract process; the cost per reaction for a commercialized eukaryotic CFPS assay kit ranges from $25 to $75	[48,51]
Operational simplicity	Simple and efficient; the preparation takes only 1–2 days	Relatively complex; the preparation process may take 4–5 days	[[49], [50], [51]]
Speed of expression	Results are available relatively quickly, within a few hours; after optimization, a yield of 200 μg/mL can be reached in about 20 min	Initial translation efficiency is low, and overall response times are long; the process typically takes 90 min to over 5 h, with a yield of only 50–100 μg/mL	[[53], [54], [55], [56]]
PTMs	PTMs unique to eukaryotes, such as glycosylation, complex phosphorylation, and acetylation, cannot be performed	Capable of performing basic PTMs such as glycosylation, disulfide bond formation, phosphorylation, and palmitoylation, it can generate more natural and functional eukaryotic proteins	[9,43,[58], [59], [60], [61]]
Protein folding and solubility	Lacking a eukaryotic molecular chaperone system, prone to forming inclusion bodies	Contains an endogenous microsomes system that promotes proper folding and increases the proportion of soluble proteins	[18,41,42]
Complex protein expression	Difficult to assemble correctly due to complex folding and multi-subunit nature of eukaryotic protein complexes	More suitable for expressing proteins requiring complex folding, such as multi-domain proteins and multi-subunit complexes	[44,45]
Codon preference	The efficiency of expressing eukaryotic genes rich in rare codons may be extremely low, necessitating codon optimization	The codon usage is closer to that of mammalian cells, resulting in better compatibility with eukaryotic genes	[62]
Endogenous pollutants	May contain endogenous proteases, nucleases, or endotoxins	Endotoxin issues are generally not present, but may contain endogenous globulins or ricin toxin	[46,[63], [64], [65]]

However, both prokaryotic and eukaryotic cell-free systems face certain limitations in practical applications. The prokaryotic CFPS system exhibits limited protein-folding capabilities, and its lack of PTM functionality prevents the execution of complex eukaryotic-specific modifications. To address this shortcoming, researchers have introduced disulfide bond isomerase C into *E. coli* systems and optimized redox conditions to achieve functional expression of complex proteins such as antibodies [47]. Eukaryotic cell-free systems also face application challenges. Unlike *E. coli*, which supports large-scale fermentation, most eukaryotic hosts require more stringent culture conditions and involve cumbersome preparation procedures [48,49]. Typically, prokaryotic cell-free systems require only a 1–2 day preparation cycle, whereas eukaryotic cell-free systems may require 4–5 days, significantly increasing system costs [50,51]. Currently, the cost per reaction for commercial eukaryotic CFPS kits ranges from $25 to $75, which is 15 to 25 times higher than that of commercially available *E. coli* CFPS systems. The use of alternative, cost-effective substrates and the simplification of preparation procedures can reduce the cost of eukaryotic CFPS systems [9]. Furthermore, compared with milligram-per-milliliter protein yields in prokaryotic cell-free systems, eukaryotic systems typically produce only 50–300 μg/mL [18,43,52,53]. To increase protein yield, researchers employed a continuous synthesis strategy in mammalian cell-free systems, boosting protein production to 1 mg/mL [42]. Meanwhile, eukaryotic cell-free systems impose stricter template requirements and exhibit slower overall reaction rates than prokaryotic cell-free systems, necessitating longer incubation times. This process typically takes between 90 min and more than 5 h, with reported protein yields of only 50–100 μg/mL [[53], [54], [55]]. In contrast, the optimized *E. coli* CFPS system can achieve a reported protein yield of 200 μg/mL in approximately 20 min [56]. To improve reaction efficiency, researchers introduced regulatory elements, such as internal ribosome entry site (IRES) sequences, to address the translation initiation limitations of proteins [44]. Additionally, automated pre-screening platforms based on eukaryotic cell-free systems have been developed for high-throughput parallel analysis of DNA templates, accelerating the transition from template design to large-scale production [57]. Eukaryotic CFPS systems have become flexible tools for complex protein expression due to their unique advantages.

## 3 Historical development of eukaryotic CFPS system

**Fig. 3:**
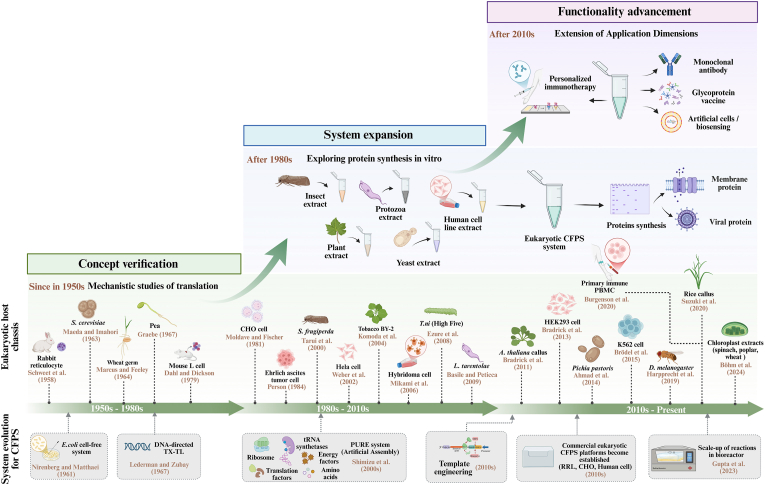
Timeline of eukaryotic CFPS system. Over its nearly 70-year evolution, the CFPS system based on eukaryotic cells has undergone three pivotal phases: concept validation, system expansion, and the current era of functional deepening. The system has expanded to encompass mainstream host platforms including mammals, plants, fungi, insects, and protozoa. Through performance optimization, integration with the PURE system and specialized human cell platforms, it has progressively advanced in therapeutic protein production, antibody and vaccine expression, and personalized medicine. Created with BioRender.com.

To establish an *in vitro* expression platform that could mimic the complex environment of eukaryotic cells, the CFPS system, using eukaryotic cells as a chassis, was explored in the 1960s. So far, in its nearly 70 years of development, it has mainly experienced three key stages: initial validation, system diversification and expansion, and performance optimization and function deepening (Fig. 3).

Earlier studies on CFPS relied more on prokaryotic cell extract, the most pioneering of which was the *E. coli* extract system, which provided a key technological framework for the exploration of eukaryotic CFPS systems. In 1961, Nirenberg and Matthaei succeeded in resolving the genetic code based on the *E. coli* extract system and established the first prokaryotic CFPS system, leading to innovative advances in *vitro* protein synthesis [66]. Subsequently, Nathans et al. accomplished the biosynthesis of phage f2 shell proteins based on this system, making *in vitro* synthesis of complete proteins a reality [67]. The transcription-translation coupling system developed by Lederman and Zubay realized DNA as a template to direct protein expression *in vitro* [68]. Despite ongoing refinements to prokaryotic CFPS systems, their inherent limitations hindered the synthesis of complex proteins. Consequently, eukaryotic CFPS systems gradually gained attention.

Initial studies of the eukaryotic CFPS system focused on understanding the fundamentals of protein synthesis in a cell-free environment. Using rabbit reticulocyte lysate (RRL), Schweet et al. first began exploring eukaryotic CFPS. They achieved *in vitro* hemoglobin synthesis using a cell-free system consisting of rabbit reticulocyte microsomes and soluble enzymes, marking the first eukaryotic CFPS system based on mammalian extracts [69]. Since then, RRL has become a classic tool for studying eukaryotic translation mechanisms and has continued to realize technological breakthroughs. Between the 1970s and 1980s, the rise of a variety of mammalian chassis, including HeLa cells, mouse L cells, Ehrlich ascites tumor cells, and Chinese hamster ovary (CHO) cells, drove the development of mammalian CFPS systems [[70], [71], [72], [73], [74], [75]]. To further expand the applications of eukaryotic CFPS systems, exploring other host chassis to build a diversified toolbox is a major research direction.

Selecting the optimal host based on the properties of the target protein and the specific application has spurred the emergence of cell-free systems derived from a wide range of sources, including plants, fungi, insects, and even protozoa. First, plant CFPS systems isolate extracts mainly from plant seeds or tissues, which have higher biosafety compared to RRL. Wheat germ is a representative chassis for the development of plant CFPS systems and was first reported in 1964 [76]. After continuous improvement, the mature wheat germ extract (WGE) system provides a powerful tool for plant virus mechanism studies and high-quality soluble protein synthesis. In addition to the efficient WGE cell-free system, an *in vitro* translation system based on various tissues was developed using pea as the model system [[77], [78], [79], [80], [81]]. The exploration of tobacco BY-2 and plant healing tissue chassis from *Arabidopsis thaliana* and rice after 2000 further addressed the issues of the source of plant raw materials and the efficiency of their preparation [[82], [83], [84]]. Second, to combine eukaryotic functionality with prokaryotic cost, fungal chassis are an ideal platform for developing economical eukaryotic CFPS systems. With proven fermentation technologies and genetic tools, cell-free platforms based on *Saccharomyces cerevisiae* were established in the 1970s [[85], [86], [87]]. With further research, *Pichia pastoris*, an important host for recombinant protein production, became a new direction for the development of fungal CFPS systems [88]. Into the 21st century, cell-free systems based on insect cells have emerged, and their utilization of baculovirus vectors has become an important platform for the production of functional proteins, such as antibodies and vaccines. Insect cells, represented by *Spodoptera frugiperda*, enabled the development of cell-free translation/glycosylation systems by virtue of their stably retained translational and post-translational components [89]. Since then, HighFive cells from *Trichoplusia ni* and *Drosophila melanogaster* cells have further expanded the sources of extracts for insect CFPS systems [90,91]. Finally, protozoa, particularly *Leishmania tarentolae*, offer a promising new host for the eukaryotic CFPS field [92]. It combines both prokaryotic mass culture and mammalian-like glycosylation capabilities, promising the synthesis of high-quality proteins while reducing cost and handling difficulties. The diversification of these host chassis has deepened the investigation of eukaryotic CFPS systems for *in vitro* protein synthesis.

In recent years, as technology has matured, research on eukaryotic CFPS systems aims to advance performance and deepen functionality. On the one hand, to better reflect the human physiological environment, researchers have expanded the use of human cell lines in eukaryotic CFPS. In addition to early Hela cells, eukaryotic CFPS systems derived from various human cell lines, such as human hematopoietic progenitor cell line K562, human embryonic kidney cell line HEK293, and hybridoma cells, have been successfully developed, which have unique advantages in synthesizing human glycoproteins for therapeutic use, antibodies, and developing antiviral drugs [[93], [94], [95], [96]]. In 2020, the application of human peripheral blood mononuclear cells (PBMC) demonstrated the exploration of the eukaryotic CFPS system toward personalized medicine [97]. Recently, plant-derived CFPS has been further evolved from conventional tissue- and cell-based extracts toward subcellular platforms, as exemplified by chloroplast extract systems established across species such as spinach, wheat, and poplar [98]. After continuous optimization, multiple commercialized eukaryotic CFPS systems have emerged, including the mammalian-derived Flexi® Rabbit Reticulocyte Lysate System (Promega), the 1-Step CHO High-Yield IVT Kit (Thermo Scientific), and the 1-Step Human In Vitro Translation Kit (Thermo Scientific). Protein yields in the Transdirect® insect cell-free system and ALiCE® plant cell-free system range from 50 μg/mL to 3 mg/mL [11,31]. On the other hand, the development of the PURE system is a new paradigm in the field of CFPS [99]. The purification of core components from cells and the *in vitro* reconstruction of translation systems with well-defined compositions help to promote the in-depth resolution of eukaryotic translation mechanisms [100]. So far, the eukaryotic CFPS system has expanded from expressing model proteins to a mature platform for synthesizing biomolecules with complex functions.

Overall, eukaryotic CFPS systems have undergone nearly 70 years of development, expanding the boundaries of synthetic biology applications. Starting from RRL as an *in vitro* translation platform for mammalian cells, it has gradually expanded the host chassis to include a wide range of cell-free systems such as plants, fungi, insects and even protozoa. In recent years, with the maturity of the technology, research has further deepened into optimization of system performance and precise reconfiguration of functions, such as the development of more efficient human cell line systems and eukaryotic PURE systems with well-defined components. Nowadays, the field of eukaryotic CFPS has entered a new stage of diversification, refinement, and high controllability, which has not only become an effective tool for basic research, but also demonstrated valuable potentials in biomedicine, synthetic biology, and other application fields.

## 4 Exploration of host chassis for eukaryotic CFPS systems

**Fig. 4:**
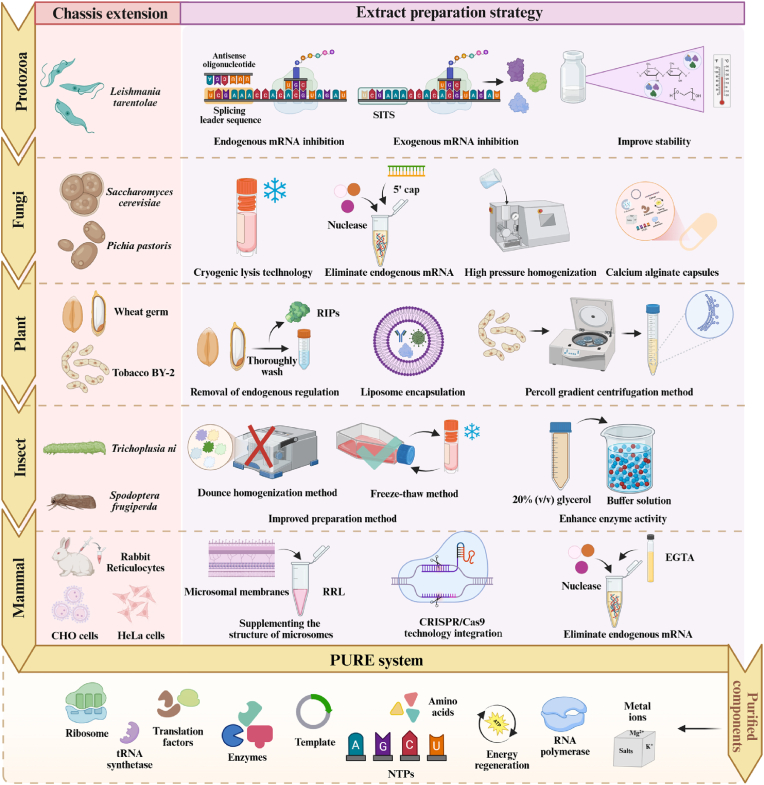
Diversification of cell platforms for eukaryotic CFPS systems. This figure illustrates the diverse chassis sources of the eukaryotic CFPS system, encompassing protozoa, fungi, plants, insects, and mammals. By rationally designing efficient extract preparation strategies tailored to different chassis characteristics, the eukaryotic CFPS platform achieves a systematic optimization chain—from chassis engineering modifications to efficient cell lysis and purification of endogenous contaminants—to maximize extract stability and activity. Beyond these extract sources, the PURE system provides a purer system composition through purified extract components, enabling more precise reaction control. Created with BioRender.com.

To meet the growing demand for complex functional proteins in biomedical research and industrial production, it is crucial to develop cell-free protein synthesis systems that combine eukaryotic translation capabilities with cost-effectiveness. However, it is difficult for a single system to accommodate diverse needs, so researchers have explored diverse CFPS platforms based on different eukaryotic chassis (Fig. 4) to achieve a balance between function and cost. Currently, the major eukaryotic host chassis come from protozoa, fungi, plants, insects, and mammals, as shown in Table 2. Although each has its own characteristics in terms of cost, translation fidelity, and modification flexibility, the conserved eukaryotic translation mechanism confers the same advantages in complex protein modification and folding as prokaryotic CFPS systems. After half a century of optimization and exploration, the development of eukaryotic CFPS systems in synthetic biology has been promoted.

**Table 2:** Comparison of different eukaryotic CFPS system.

Chassis cell	Strengths	Weaknesses	Applications	References
**Protozoan extracts**				
*Leishmania tarentolae*	1. Rapid growth suitable for large-scale cultivation 2. Non-pathogenic, low cost and accessible 3. Fast and easy preparation of extracts 4. Mammalian-like complex N-glycosylation. 5. Unique genetic properties can reduce background interference	1. Low protein yields to meet large-scale production needs 2. Unstable system activity with significant batch-to-batch variation 3. Unclear PTM type 4. Time-consuming post-transfection screening, possible morphological transformation 5. Limited adoption and low technical maturity	1. High-throughput analysis of protein interactions 2. High-quality expression of complex post-translationally modified proteins such as human membrane proteins, antibodies and enzymes 3. Lyophilization technology for portable devices	[19,34,46,92,101,102,[108], [109], [110], [111], [112], [113],^183^,^184^]
**Fungal extracts**				
*Saccharomyces cerevisiae*	1. Abundant strain resources and mature genetic tools 2. Simple and inexpensive cultivation for fast preparation of extract 3. Presence of yeast microsomes facilitates secretory protein translocation 4. Correct protein folding and glycosylation modification	1. Lower protein yield compare to prokaryotic 2. Lack of mammalian-like PTMs	1. Exploring eukaryotic translation mechanisms to support the design and production of eukaryotic ribosome-associated drugs and unnatural proteins 2. Production of human protein therapeutic agent 3. Viral protein expression and VLPs assembly 4. Metabolic engineering and bioenergy Manufacturing	[115,116,118,125,176,177,[185], [186], [187], [188], [189]]
*Pichia pastoris*	1. Ability to grow to high cell densities with high volumetric productivity 2. Genetic tractability allows precise control of target proteins 3. Yeast microsomes are available 4. Human N-glycosylation and disulfide bond formation are possible	1. Inefficient expression 2. Limited applications still need to be further explored	1. Production of humanized therapeutic protein 2. Synthesis of Hepatitis B VLPs 3. Generation of functional ion channels and site-specific modifications of GPCRs	[9,17,114,117,128,129]
**Plant extracts**				
Wheat germ	1. The highly researched and technologically mature, commercially available system ALiCE® has been developed 2. Excellent eukaryotic translation efficiency and high-throughput analysis capability 3. Ability to form disulfide bonds to express high quality soluble proteins 4. Potential for selective translation initiation and protein PTMs, such as phosphorylation	1. Time-consuming and costly preparation of cell extracts 2. The absence of endogenous membrane machinery precludes natural glycosylation. 3. Limited PTMs	1. Basic mechanism investigation 2. Expression of high quality soluble proteins, including membrane proteins, antibodies and viral proteins. 3. Antibody serology high-throughput screening for vaccine and drug development 4. Structural biology and proteomics analysis, such as protein microarray design 5. Artificial cell construction and biosensing assays.	[10,32,61,133,134,[138], [139], [140], [141],[190], [191], [192], [193], [194], [195], [196], [197], [198]]
Tobacco BY-2	1. Fast and easy experimental processes enable high potential for scale-up 2. Presence of microsomes allows disulfide bond formation and PTMs such as glycosylation 3. Higher protein yield than commercial WGE formulations 4. Linear non-destructive amplification for industrial and production requirements	1. Limited research compared to WGE 2. Application scenarios still need to be expanded	1. Investigating the mechanism of translational replication of plant RNA viruses 2. Synthesis of functionally active proteins, including glucose oxidase, antibodies, membrane proteins 3. Synthesize and screen HBc VLP vaccine candidates 4. Act as metabolic factories to produce high value natural products and secondary metabolites	[18,31,82,135,142,[199], [200], [201], [202], [203]]
Rice callus	1. Tissue culture materials are readily available 2. Easy to genetically modify to customize and improve system performance	1. Immature development and lack of systematic optimization 2. Narrow range of applications with potential to be tapped	1. Elucidation of molecular mechanisms in the eukaryotic translation system 2. Functional protein synthesis, such as human hERG potassium channels 3. Allows multi-gene expression and metabolic engineering, supports 2A peptide-based double *cis*-trans mRNA expression	[81,84,132,143]
**Insect extracts**				
*Spodoptera frugiperda 21*	1. Easily cultured for large-scale extract preparation 2. Endotoxin-free, avoiding chemical pre-treatmen 3. Highly sensitive to baculoviruses with high protein expression capacity. 4. Endogenous microsomal structure supports transmembrane translocation and PTM function of proteins. 5. Commercialization Transdirect® insect cell-free system now available	1. Low protein yield compared to live cell systems 2. Cost ineffective and difficult to establish 3. Limited application	1. Supporting correct folding and functional studies of membrane proteins such as EFGR and GPCR 2. Production and modification of functional antibody fragments 3. Specific doping of ncAA 4. Expression of UPOs, expanded enzyme libraries and protein engineering	[11,43,89,[144], [145], [146], [147], [148], [149], [150], [151],[204], [205], [206], [207], [208], [209], [210]]
*Trichoplusia ni* (HighFive)	1. Low cost of extract preparation 2. Possesses PTM ability and microsomal structure similar to Sf21. 3. Glycosylation pattern is closer to that of mammalian cells.	1. Limited research	1. Synthesizes active proteins such as luciferase and beta-galactosidase. 2. Suitable for large-scale protein production and functional studies.	[90]
*Drosophila melanogaster*	1. Clear genetic background for mechanistic studies. 2. Capable of DNA damage sensing and phosphorylation signaling.	1. Limited protein production applications 2. Limited research	1. Probing DNA repair mechanisms and signaling pathways. 2. Efficiently recombine complex chromatin for the synthesis of complex nuclear and chromatin-associated proteins. 3. Cell-free genomics, which probes the interaction of TF with chromatin.	[91,211,212]
**Mammal extracts**				
Rabbit reticulocyte	1. Long-established, mature and well-established system 2. Advantages in humanoidization of PTM 3. Commercialized system such as Promega Flexi® system	1. Extract preparation is cumbersome and involves complex animal tissue processing 2. Low protein yield 3. Lack of endogenous microsomes, exogenous additions required to support PTM	1. Exploring eukaryotic translation mechanisms 2. Analyzing ribosome function to uncover disease-related mechanisms 3. Resolving membrane protein topologies 4. Studying drug resistance mechanisms 5. Phosphorylated protein expression 6. Viral assembly simulation 7. Production of viral proteins and VLPs, such as HBc and HIV	[65,69,[152], [153], [154], [155], [156], [157], [158], [159], [160], [161], [162], [163],[213], [214], [215], [216], [217], [218], [219], [220]]
Chinese hamster ovary	1. Well-known and characterized cell line 2. Contain nature microsomal structures 3. Mammalian PTMs, suitable for humanized protein expression	1. Higher culture costs and operational complexity 2. Low protein yield compared to prokaryotic cell-free systems	1. Antibody expression and engineering research 2. Synthesis of membrane proteins and glycosylated proteins, such as EFGR and EPO and ion channel 3. Supporting assessment of toxin activity and characterization of viral structure 4. Synthesis of multiple vaccine antigens for vaccine design	[8,[42], [43], [44], [45],^49^,^72^,^74^,[164], [165], [166], [167], [168], [169], [170], [171], [172],[221], [222], [223], [224], [225]]
Human cell lines	1. System technology is mature with commercially available kits such as the 1-Step Human In Vitro Translation Kit 2. Contains endogenous microsomal structures to support optimal environment for folding and assembly of human PTMs 3. Humanized protein folding and assembly	1. Higher culture costs and operational complexity 2. Low protein yield compared to prokaryotic cell-free systems	1. Synthesis and analysis of functional membrane proteins, such as the nuclear membrane protein SUN1/2 2. Expression of viral proteins and VLPs, analyzing viral mechanisms to promote antiviral drug development 3. Human glycoprotein synthesis	[7,55,62,[94], [95], [96], [97],^173^,^174^]
**PURE system**	1. Components are well defined and highly controllable 2. Lack of proteases and nucleases reduces losses 3. Manipulation of the translation machinery to solve eukaryotic-specific translation problems 4. Genetic code expansion or reprogramming	1. Complex and costly preparation process 2. Eukaryotic systems are less well studied 3. low protein yield 4. Limited application	1. Modeling eukaryotic translation mechanisms 2. In vitro remodeling of subcellular organelles	[2,[175], [176], [177], [178], [179], [180], [181], [182]]

### 4.1 Protozoan extracts

Protozoa are fast-growing and easy to cultivate on a large scale by fermentation, which makes their rapid growth characteristics an ideal source for producing cell-free extracts [101]. Compared to other eukaryotes, protozoan extracts are easy to prepare and have unique genetic properties and N-glycosylation capabilities similar to those of mammals [92,102]. Conserved splice leader sequences on endogenous mRNAs reduce background interference, improving the purity of target protein expression [46]. Due to these unique physiological and biochemical properties, protozoa have been used in the development of CFPS systems to improve the quality and efficiency of protein expression while reducing the difficulty and cost of manipulation for biomedical research and applications.

A variety of protozoan chassis have been used as research models to understand protein synthesis in a cell-free environment. Early in the development of the CFPS system, researchers used ribosomal cell-free systems isolated from protozoa to explore protein synthesis mechanisms. Among them, protozoan chassis, represented by *Tetrahymena pyriformis*, *Crithidia oncopelti*, *Paramecium aurelia*, *Entamoeba histolytica*, and *Rumen protozoa*, were used to systematically study the construction of cell-free systems [[103], [104], [105], [106], [107]]. To obtain a highly active cell-free system, several cell lysis methods were evaluated. The glass bead fragmentation method proved optimal, yielding extracts that not only contained polysomes, but also possessed the highest specific activity with regard to amino acid activation [104]. Furthermore, the exploration of protein synthesis pathways and the determination of optimal reaction conditions, not only offer a crucial model for understanding the protein synthesis mechanism in protozoa, but also lay the groundwork for the future development of efficient and stable protozoa cell-free expression platforms [104,106,107].

Advances in *vitro* protein synthesis techniques have prompted researchers to explore protozoan chassis better suited to high-yield recombinant protein expression. *L. tarentolae* became a central representative in the field of protozoan CFPS [92]. The performance of the CFPS system based on *L. tarentolae* extract (LTE) was effectively enhanced by the key features and regulatory mechanisms of this substrate. *L. tarentolae* endogenous mRNA has a common splicing leader sequence, and the background expression of the endogenous gene was effectively suppressed by antisense oligonucleotides [46]. At the same time, by combining with species-independent translation initiation sites (SITS), the translation efficiency of the exogenous gene was significantly enhanced, and up to 300 μg/ml of recombinant protein could be produced within 2 h [108]. Additionally, LTE preparation is a key factor in determining system efficiency, and its quality is affected by cell density, fragmentation methods, and purification steps. Harvesting *L. tarentolae* cells at the logarithmic growth phase can yield optimal protein expression levels. Meanwhile, the use of a high-pressure nitrogen cell crusher to break the cells and a gel filtration column to remove impurities proved to be an efficient protocol for LTE preparation, minimizing cell damage and improving the purity and activity of the lysates [109]. Adjusting the LTE addition ratio within the range of 20% to 60% balances protein expression levels and protein aggregation phenomena, thereby enhancing the stability and product accuracy of the LTE CFPS system [110]. However, the storage and stability of extracts remain critical challenges for the CPFS system. The incorporation of lyoprotectants such as polyethylene glycol and trehalose during the drying process enhanced the stability of LTE at room temperature [111]. Through chassis optimization, protozoa have become a mature host source for eukaryotic CFPS systems.

With N-glycosylation capabilities comparable to mammals, protozoa hold significant value for expressing human membrane proteins, antibodies, and enzymes, while delivering higher-quality proteins than the *E. coli* system [34,46,102,[110], [111], [112], [113]]. However, these cell-free systems still suffer from many shortcomings, including unstable system activity, high sensitivity to magnesium ion concentration, significant batch-to-batch variation, and unclear types of post-translational modifications, leading to their limited versatility [19]. In the future, the development of the protozoan cell-free system needs to focus on two core directions: scale-up and modification capability. Solve the process challenges of large-scale lysis production and increase the yield of recombinant proteins to meet the demand of industrialized production. Explore in depth and expand the types of post-translational modifications in the system, such as phosphorylation and acetylation, so that it can produce complex proteins with structures and functions closer to the natural state, and thus play a more important role in biomedicine and synthetic biology.

### 4.2 Fungal extracts

Developing cost-effective eukaryotic CFPS systems is crucial for large-scale protein production. As a well-established fungal model organism, yeast has well-developed genetic manipulation techniques and, similar to *E. coli*, can be cultured rapidly and inexpensively in bioreactors or shake flasks [114,115]. Moreover, yeast extracts are not only straightforward to prepare but also contain microsomes that facilitate protein translocation and complex PTMs, enabling high levels of recombinant protein expression [9,[116], [117], [118]]. Therefore, yeast may provide a more economical host source for eukaryotic CFPS systems, balancing “eukaryotic function” with “prokaryotic cost”. Currently, *S. cerevisiae* and *P. pastoris* are the primary sources of yeast extracts and are the most widely used species in research on fungal cell-free systems.

Due to its abundant strain resources and mature genetic tools, *S. cerevisiae* has become the preferred fungal chassis for cell-free research. Earlier yeast extracts were prepared by lysing spheroplasts or whole cells, which was a cumbersome and costly process [119]. The mechanical lysis procedure simplifies the extract preparation protocol, while eliminating cumbersome processing steps and costly reagents enhances the economic viability of the yeast CFPS system [115]. The extraction protocol incorporating cryogenic lysis technology further enhances the performance of the constructed yeast CFPS system [120]. Moreover, optimization of yeast fermentation conditions significantly enhanced extract activity, resulting in a fourfold increase in protein synthesis yield [121]. However, to enhance the expression of exogenous templates, resolving endogenous background suppression is crucial. By employing micrococcal nuclease to eliminate endogenous mRNA and adding 5′-cap structure analogues for competitive inhibition, the system becomes strictly dependent on exogenous templates, thereby substantially enhancing translation fidelity [[122], [123], [124]]. As the technology continues to mature, the CFPS platform based on *S. cerevisiae* not only contributes to understanding translation mechanisms but also demonstrates significant value in the field of biomanufacturing. Encapsulating yeast lysate within calcium alginate capsules effectively resolves substrate/product inhibition issues in conventional simultaneous saccharification and fermentation, offering a novel approach for large-scale bioethanol production [125]. This encapsulated CFPS system expands the toolkit for bioenergy manufacturing.

Notably, *P. pastoris* (syn. *Komagataella* spp.) is an important host for the production of recombinant proteins, which is gradually increasing its influence in the study of cell-free systems. First, instead of limiting themselves to using wild-type strains, researchers are empowering cell-free systems at the source by means of genetic engineering [88]. Highly active yeast prototype strains were developed by evaluating the effects of different single-knockout yeast strains on CFPS activity [126]. By designing ribosome-enriched *P. pastoris* strains, a significant increase in translational machinery abundance in extracts was achieved. Concurrently, ribosome biosensors were employed to determine the harvest time point with the highest intracellular ribosome levels, enabling dynamic optimization of extract preparation [114,127]. Furthermore, to address protease-mediated product degradation in cell-free systems, protease-deficient strains such as SMD1163 were used for extract preparation. This approach effectively preserved the integrity of the synthesized protein, yielding approximately 50 μg/ml of sfGFP during a 5-h batch reaction [53]. Second, the method of cell lysis significantly impacts the activity of yeast extracts. Compared to the relatively simple sonication, high pressure homogenization is a more effective lysis method for releasing intracellular contents and achieving high-yield extracts [117]. Through continuous development, the *P. pastoris* CFPS system has also gradually expanded its application from the expression of model proteins to the synthesis of biomolecules with complex functions. With its potential to mimic the human N-glycosylation pathway, the system has been used to efficiently express therapeutic proteins with biopharmaceutical value [17,128,129]. Meanwhile, its rapid response and miniaturization make the *P. pastoris* CFPS system a platform for high-throughput screening and prototyping, which is expected to be used for the rapid screening and validation of new metabolic pathways, gene components, or drug targets, thus shortening the cycle from design to validation.

In general, fungal cell-free systems are undergoing rapid development from useable to versatile. Through strain engineering and innovations in preparation methods, yeast cell-free systems have emerged as reliable and cost-effective solutions for protein synthesis. While the presence of endogenous yeast microsomes facilitates correct protein folding and PTMs, there remains significant room for improvement in protein expression efficiency. Furthermore, although the engineered humanized N-glycosylation pathway in yeast provides a robust foundation for glyco-engineering and the production of therapeutic glycoproteins, the potential of yeast CFPS systems needs further exploration. Beyond the unicellular fungus yeast, extracts derived from other fungi, such as *Candida albicans* lysates or filamentous fungal lysates from *Neurospora crassa* and *Aspergillus niger*, have also been developed for CFPS applications to aid in biochemical analysis of the translation machinery and the synthesis of complex proteins [130,131]. However, relevant literature is scarce, indicating that these systems are not yet mature. Future research on fungal cell-free systems need to focus on expanding the range of fungal chassis, improving expression efficiency, and enhancing compatibility with different protein types. These advancements will enable the synthesis of a wide range of functional proteins, thereby increasing the system's value in biomanufacturing.

### 4.3 Plant extracts

Plant extract-based CFPS systems are important tools for *in vitro* expression of eukaryotic organisms and offer unique advantages for expressing complex eukaryotic proteins. First, plant materials are flexible in origin, easy to genetically modify, and possess unique physiological research value [132,133]. Second, plant extracts not only provide a complex protein-processing environment not available in prokaryotic systems, but are also more accessible and biologically safer than other mammalian hosts [18,134,135]. The uniqueness of different plant species or tissues can enhance the customization ability of eukaryotic CFPS systems, which are highly application-oriented [84]. Therefore, it is valuable to explore the application of plant chassis in CFPS. Currently, wheat germ and tobacco BY-2 are the main plant chassis for exploring the capacity of plant CFPS systems. Other plant sources, such as rice, pea and *A*. *thaliana*, have reinvigorated the plant cell-free system.

The wheat germ-based cell-free system has been developed over nearly 60 years to become a mature platform for CFPS. The early reported WGE cell-free translation system relied on radioactive amino acids detected in isolated wheat germ ribosomes to catalyze the synthesis of peptide components [76]. However, higher plants contain ribosome-inactivating proteins (RIPs) such as ricin toxin, which prevent the proper binding of elongation factors by disrupting the α-sarcin/ricin loop region on the large subunit rRNA of eukaryotic ribosomes, thereby inhibiting protein synthesis [63,64,136]. Thoroughly washing wheat germ to remove endosperm contaminants eliminates endogenous inhibitors, effectively enhancing the stability and activity of the WGE *in vitro* translation system [50]. Nevertheless, in traditional laboratory protocols, preparing 6 mL of extract typically requires processing approximately 6 kg of wheat seeds, making the handling of wheat germ particles cumbersome [48]. Compared to small-scale liquid nitrogen grinding procedures, utilizing food processing grinders/blenders such as the Waring Blender for pulverization accommodates larger-scale raw material processing, significantly enhancing operational efficiency. The modified WGE preparation protocol yielded 5 ml of highly active extract in 4-5 days [137]. With continuous improvement, the WGE CFPS system, with its optimal eukaryotic protein expression efficiency, is widely used to synthesize a wide range of high-quality soluble proteins, including protein complexes, membrane proteins, viral proteins, antibodies, and other structurally complex functional proteins, to support vaccine screening and drug discovery [10,61,[138], [139], [140], [141]]. The WGE cell-free system has become a valuable platform for cross-disciplinary applications in cutting-edge fields such as synthetic biology and bioelectronics.

Tobacco BY-2 serves as the CFPS system chassis, offering an alternative solution to address the cumbersome and time-consuming core issues inherent in preparing traditional plant-derived cell-free platforms. Tobacco BY-2 extracts (BYE) containing endoplasmic reticulum were obtained by removing protoplast vesicles using Percoll gradient centrifugation, thus constructing a new system based on plant cell extracts [82]. Tobacco BY-2 allows for continuous suspension cultures, avoiding the limitations of WGE that require large quantities of wheat seed and storage. The simple cell lysis procedure enables the preparation time of BYE to be drastically shortened to 4-5 h, which greatly improves the experimental efficiency [142]. Through systematic optimization, the performance and practicality of the BYE cell-free system have been significantly enhanced. Compared with commercial WGE formulations, BYE yields higher protein levels in both batch and dialysis reaction modes [135]. The commercial version of BYE has achieved high-yield production at 3 mg/mL [31]. Moreover, due to the presence of actively translocating microsomal vesicles originating from the endoplasmic reticulum, BYE exhibits advantages in PTMs. By expressing eYFP proteins with N-terminal melittin signal peptide, it was verified that the target proteins could be translocated into endoplasmic reticulum microsomes in BYE. Concurrently, glycosylation and disulfide bond formation are feasible in the BYE CFPS system, enabling the synthesis of functional full-size antibody M12 [18]. Leveraging its robust eukaryotic protein expression capabilities, the BYE cell-free system is not only utilized for the rapid expression and screening of vaccine candidates but also demonstrates invaluable potential in the production of secondary metabolites.

In addition to the common WGE and BYE cell-free systems, other plant chassis, including peas, Arabidopsis, and rice, can also be employed for constructing cell-free systems. Peas serve as a classic plant material from early research, offering diverse tissue sources. Pea shoots, pea fruits, pea cotyledons, and dry pea primary axes are all used in the preparation of pea extracts [[77], [78], [79], [80], [81]]. Furthermore, as a model organism for botanical research, *A*. *thaliana* possesses rich genetic resources. An *in vitro* translation system based on *A*. *thaliana* seedling callus was established using extracts prepared by the Percoll gradient centrifugation method [83]. However, as in pea and *A. thaliana*, rice material derived from seed or embryo sources is limited by the growth cycle [143]. The development of the CFPS system based on rice callus extracts (RCE) exemplifies the transition from traditional tissue culture to modern biotechnology. Callus tissue can be mass-produced through tissue culture techniques, making rice raw materials more readily available [84]. Furthermore, efforts have expanded toward subcellular-level cell-free systems, successfully yielding transcriptionally and translationally active chloroplast crude extracts from a monocot crop (wheat), a dicot herbaceous plant (spinach), and a dicot tree (poplar). By optimizing protocols for chloroplast isolation, lysis, and extract preservation tailored to different plant tissues, the study demonstrated that this subcellular cell-free preparation strategy exhibits a high degree of cross-species versatility [98]. These diverse plant chassis present new opportunities for cell-free systems. The RCE CFPS system facilitates the establishment of customizable CFPS platforms targeting multiple protein targets. Combined with the well-established CRISPR/Cas9 or TALEN genome-editing technologies in rice, it is expected that customized CFPS platforms targeting specific protein expression or PTMs will be constructed in the future [132].

The plant CFPS system has become an important research method in life sciences and biotechnology due to its unique advantages for expressing complex proteins. After decades of development, the systems represented by wheat embryos and tobacco BY-2 cells have become increasingly mature, while novel plant chassis such as rice, pea, and *A*. *thaliana* also show great potential to enrich the CFPS toolbox. Based on different chassis characteristics, the mainstream cell-free platform has been systematically optimized, significantly enhancing its performance and practicality. The production of complex proteins and the synthesis of secondary metabolites have demonstrated the value of plant systems in biomanufacturing and drug discovery. Despite significant progress, plant cell-free systems still face several challenges that limit their broader application. The relatively cumbersome process of preparing high-purity wheat germ or other plant cells limits their use for low-cost, large-scale production. Although linear scale-up of bioreactors has been achieved, the yields and cost-effectiveness for large-scale industrial production are still not comparable to the highly optimized *E. coli* system [31]. In addition, the type, efficiency, and fidelity of PTMs are still far from those of mammalian cells. The stability and versatility of the new chassis currently fall well short of those of the mature WGE and BYE systems. In the future, analyzing metabolites and inhibitory factors in plant extracts and establishing a component-function relationship database will help maintain the efficiency and persistence of reaction systems. Moreover, deep genome editing of plants with clear genetic backgrounds, such as rice, enables the enhancement and reconstruction of endogenous modification pathways and the design of super chassis from scratch. Finally, the development of continuous flow bioreactors suitable for plant cell-free systems enables the integration of automation and scale-up production technologies to meet the needs of industrial-scale applications. Through multidisciplinary crossover and technological innovation, efficient and flexible plant cell-free platforms will be invaluable in fields such as biomedicine, materials science and energy development.

### 4.4 Insect extracts

Insect cells infected with baculovirus are effective platforms for functional protein synthesis, combining the complex post-translational modification capabilities of eukaryotic expression systems with the high efficiency of prokaryotic expression systems, and have been used to develop cell-free systems. It uses baculoviruses as gene vectors to insert target genes downstream of a potent polyhedrin promoter, thereby achieving high-level expression of target proteins through the viral superexpression capacity. Moreover, the insect cells possess PTM abilities unique to eukaryotes, such as glycosylation, phosphorylation, lipidation, signal peptide cleavage, and disulfide bond formation. The endoplasmic reticulum component was retained in its prepared extracts, which can form active microsomal structures that support signaling peptide-mediated transmembrane translocation. With its excellent protein expression capabilities, *S. frugiperda* is the preferred choice for preparing insect extracts. In addition, *T*. *ni* and *D. melanogaster* cell lines have also been used for field-specific studies.

*Spodoptera frugiperda* 21 (Sf21), based on baculovirus infection, serves as the primary source for constructing insect CFPS systems. However, high-pressure preparation may be the primary cause of disruption of post-translational modifications in insect extracts (ICE). To enhance the translation efficiency of ICE-based CFPS, the Mini-Bomb cell disruption chamber was employed to effectively preserve translational and post-translational components through high-pressure nitrogen treatment [144]. However, this standardized preparation process for ICE is time-consuming and requires support from expensive instruments [145]. The French press method replaces the costly Mini-Bomb cell disruption chamber while eliminating the chromatographic separation step, preserving glycosyltransferases in the cell extract and significantly reducing both cost and time [146]. Additionally, HighFive cells from *T*. *ni* are also used for ICE preparation. In contrast to the conventional Dounce homogenization method, the *T*. *ni* cell-free system employs a freeze-thaw approach. This further optimizes the ICE preparation protocol by stabilizing intracellular enzyme activity through the addition of 20% (v/v) glycerol to the extraction buffer [90]. This approach also works with Sf21 insect cells, where optimized component concentrations yield approximately 45 μg of luciferase per milliliter of reaction system. Through continuous refinement, the ICE-based CFPS system has become a valuable platform for modern protein engineering and functional analysis. The mild preparation allowed the retained ER component in ICE to form a microsomal structure with translocation activity, supporting signal peptide-mediated protein transmembrane translocation and a variety of PTM functions [43,[147], [148], [149]]. Beyond biomanufacturing, *D. melanogaster* embryo extract (DREX) possesses DNA damage-sensing and phosphorylation-signaling capabilities, expanding the application of the ICE-based CFPS system in cell-free genomics [91].

Over the years, insect cell-free systems have been expanded on a variety of chassis cells, including Sf21, *T. ni*, and *D. melanogaster* embryos. The yield of commercial insect CFPS systems typically reaches 50 μg/mL [11,150]. Although ICE-based optimized systems have demonstrated improved efficiency, achieving up to 285 μg/mL, the overall protein yield remains limited compared to live-cell platforms [43,151]. Despite these yield constraints, the system retains value for expressing complex proteins, including membrane proteins and antibodies. Furthermore, the scalability of the insect CFPS system still offers significant potential for optimization, including editing the host chassis, optimizing reaction conditions, and adding new cofactors. Moving forward, improving the yield-to-cost ratio remains a major issue that needs to be addressed urgently for this system. At the same time, the combination of automated reaction flow and improved high-throughput screening capability can help expand its application in drug discovery and vaccine development. By integrating microelectronic or micro-optical sensors and actuators, the automated platform enables online monitoring of reaction parameters. The cross-fertilization of multiple disciplines, including materials science and electrical engineering, is expected to facilitate the long-term development of ICE-based CFPS systems.

### 4.5 Mammal extracts

Compared to other host platforms, the mammalian CFPS system offers distinct advantages in humanized PTMs, demonstrating exceptional capability in synthesizing humanized proteins. It enables complex PTMs such as glycosylation, phosphorylation, and disulfide bond formation, making it widely used for synthesizing complex therapeutic proteins and studying translational mechanisms. The natural microsomal structure supports the transport, embedding, and proper folding of membrane proteins, making it an ideal tool for expressing complex membrane proteins. To date, rabbit reticulum cells, CHO cells, and various human cell lines are the primary sources of mammalian extracts.

Rabbit reticulocytes serve as a mature chassis for constructing eukaryotic CFPS, achieving technological breakthroughs from fundamental translational mechanism research to complex protein synthesis. RRL was derived from reticulocytes of rabbits injected with acetylphenylhydrazine-induced anemia. The first RRL-based eukaryotic CFPS system demonstrated *in vitro* hemoglobin synthesis by radioactive amino acid labeling [69]. Similar to yeast extract, endogenous mRNA was removed from RRL using micrococcal nuclease to reduce background interference. Addition of the selective chelator EGTA inactivated the residual nuclease without affecting the translation of the exogenous mRNA [65]. However, ribosome-associated free energy-consuming metabolic activities in RRL suppress protein production by competing for energy supply. Reducing the ribosomal component ratio of RRL increased ATP availability [152]. Additionally, to address the lack of endogenous microsomal structures in RRL, exogenous supplementation with canine pancreatic microsomal membranes enabled the proper folding and PTMs of secretory and transmembrane proteins, such as glycosylation and disulfide bond formation [153,154]. Compared to the WGE system, high concentrations of canine pancreatic microsomal membranes did not significantly inhibit the translational activity of the RRL system [155]. As a mature mammalian cell-free system, the RRL CFPS platform not only enables in-depth analysis of eukaryotic translation mechanisms and membrane protein topologies but also reveals drug resistance mechanisms and expresses functional proteins with PTMs [[156], [157], [158], [159], [160], [161], [162], [163]]. These applications highlight the significant value of RRL in fundamental biological research, the exploration of disease mechanisms, and the analysis of complex protein assembly.

CHO cell, as an alternative chassis to RRL, has evolved into an excellent cell-free mammalian system. The initial preparation of CHO extracts employs the RRL-identical micrococcal nuclease treatment, reducing background translation effects and enabling efficient translation of both natural and synthetic mRNA templates [72,74]. The EGTA chelation strategy for calcium ions protected the extracted proteins from degradation by endogenous proteases and enhanced lysis efficiency [164]. However, like other mammalian platforms, low protein yield remains a limitation of the CHO cell-free system. First, stress during cell lysis induces phosphorylation of eukaryotic initiation factor 2 (eIF2). Exogenous supplementation with vaccinia virus protein K3L and human protein GADD34 improved eIF2 phosphorylation, boosting yields by 100-fold [8,49]. Second, engineered modifications to CHO cells further enhanced system performance. Transfection of CHO cells expressing the eIF2α-S52A mutant, which resists eIF2α phosphorylation, yielded extracts with 3.4-fold increased translation efficiency [165]. Furthermore, CRISPR/Cas9 technology integrated the T7 RNA polymerase gene into the Rosa 26 site of the CHO genome, eliminating the need for exogenous T7 RNA polymerase in cell-free reactions [166]. Beyond addressing yield bottlenecks, PTMs of functional proteins are also a key focus in mammalian cell-free systems. Compared to RRL, CHO extract possesses a natural microsomal structure that supports mammalian PTMs. Increasing the microsomal fraction in CHO extracts or employing iodoacetamide pretreatment significantly enhances the capacity for glycosylation and disulfide bond formation [167,168]. Leveraging the translocation properties of microsomal structures within the extract, the CHO cell-free system has become an ideal tool for the synthesis of membrane proteins, glycosylated proteins, and virus-associated proteins, as well as for antibody engineering research [42,45,[169], [170], [171], [172]].

Nowadays, various cultured human cell lines (cHCLs) are increasingly used as host sources for developing CFPS systems. The HeLa cell line, with its long history of research, serves as a crucial model for establishing cHCL-based CFPS systems, where protein synthesis is activated by supplementing hemin [70,173]. Moreover, the human hematopoietic progenitor cell line K562 and the human embryonic kidney cell line HEK293, were also developed for the construction of the CFPS system [95,96]. However, translation initiation is the rate-limiting step in protein synthesis. Direct supplementation of translation initiation factors, such as eIF2, eIF2B, and eIF4, into HeLa extracts can significantly enhance protein synthesis [55]. To address eIF2α phosphorylation, the same strategy employed for CHO cells was adopted: supplementation with K3L and GADD34 proteins effectively relieved translational repression [94]. Concurrently, HEK293 cells were engineered for endogenous expression of GADD34 and K3L proteins, thereby optimizing the cHCL extract preparation protocol [174]. As the system continued to be refined, simple cHCL cell-free system construction procedures were reported, allowing for in-laboratory completion [7]. Commercially available kits based on HeLa cell extracts can yield 100 μg/mL of protein in just 90 min. In addition to the typical cHCL, Ehrlich ascites tumor cells, hybridoma cells, mouse L cells, and PBMCs are also employed in specific cell-free studies [71,73,75,93,94,97]. These systems exhibit microsomal characteristics similar to those of CHO CFPS systems, enabling diverse protein PTMs. They not only facilitate the functionalization of full-length membrane proteins and the synthesis of virus-like particles but also provide an alternative platform for antiviral drug development and the synthesis of therapeutic human glycoproteins and antibodies [62,94].

Since its development in the 1950s, the mammalian cell-free system has undergone technological breakthroughs spanning basic research on the translation mechanism to complex protein synthesis. However, due to the complexity of the translation machinery, mammalian cell-free systems typically exhibit lower expression efficiency compared to bacterial cell-free systems. Protein yields generally range around 50 μg/mL, with maximum yields reaching nearly 1 mg/mL [42]. In addition, commercial RRL systems are expensive and require supplementation with exogenous microsomes, making them economically inefficient. Meanwhile, the mammalian extract preparation and manipulation process is complex, which limits its scale-up applicability. In the future, simplifying the preparation and operation process of this system and developing a high-throughput screening platform will help promote its greater role in drug screening, vaccine design, and antibody engineering.

### 4.6 PURE system

The PURE system represents a significant advancement in the CFPS field, transitioning from crude extract systems to well-defined component systems. By precisely defining 36 purified components, including ribosomes, translation factors, tRNAs, and energy systems, this system eliminates interference from nucleases, proteases, and endogenous mRNA present in crude extracts [175]. Compared to traditional crude cell extracts, the PURE system offers high controllability and flexibility, allowing independent optimization of component concentrations to maximize protein expression according to specific requirements [2].

The formative evolution of cellular chassis has shifted from complex crude extracts to well-defined reconstituted systems. While extensive research exists on the *E. coli*-based PURE system, eukaryotic-specific translation mechanisms still require dedicated eukaryotic systems. As a prototypical eukaryotic CFPS host, *S*. *cerevisiae* has pioneered component mechanistic studies to advance eukaryotic PURE systems [100]. Building upon overcoming translation initiation constraints in yeast 80S ribosome for *in vitro* reconstruction, the addition of yeast S100 extract to yeast tRNA mixtures enabled aminoacyl-tRNA synthesis, providing essential components for translation elongation. Further integration of elongation factors eEF1A, eEF2, and eEF3, termination factors eRF1 and eRF3, and recycling factors Rli1, Hbs1, and Dom34 from yeast and *E. coli* facilitated the construction of an *in vitro* reconstituted translation system [[176], [177], [178]]. Additionally, purified yeast components were employed to investigate the *in vitro* reconstruction of organelle translation systems. Building upon a heterologous yeast tRNA pool, purified mitochondrial translation factors, including IF-2 mt, IF-3 mt, EF-Tumt, EF-Tsmt, EF-G1 mt, EF-G2 mt, RF-1 Lmt/mtRF 1a, and RRFmt, along with 55S mitochondrial ribosomes, were incorporated to reconstruct an *in vitro* mammalian mitochondrial translation system [179]. This reconstituted system provides a viable model for studying genetic disorders associated with mitochondrial translation defects. Although the design of eukaryotic chassis components remains exploratory, the newly developed OnePot PURE system offers a streamlined strategy for component production. This integrated purification technology performs comparably to expensive commercial systems like PURExpress, enabling production of all PURE system components within one week while avoiding RNA degradation and protease contamination inherent in traditional ribosome purification [[180], [181], [182]]. Consequently, this streamlined preparation method may offer an alternative for developing and iterating PURE systems from diverse sources.

The PURE system, characterized by its well-defined components and open, controllable nature, has become a vital tool for elucidating biological processes and synthesizing synthetic biological proteins. Current research is transitioning from prokaryotic models to eukaryotic chassis, advancing fundamental scientific areas such as simulating eukaryotic translation mechanisms and reconstructing mitochondrial systems. However, the development of eukaryotic PURE systems remains constrained by the complexity and high cost of component preparation, as well as lower protein yields than those of established crude extract systems. Future research should broaden the sources of eukaryotic chassis and deepen the intelligent design of system components to optimize stability and yield, thereby advancing their widespread application in drug design, protein production, and precision medicine.

## 5 Design of core components and reaction environment for the eukaryotic CFPS system

The development of extracts from multiple eukaryotic hosts has advanced CFPS systems. In the CFPS reaction, transcription and translation of exogenous templates require not only extract inputs but also additional additives. Compared to extract preparation, which is influenced by distinct eukaryotic host characteristics, the design of templates, energy regeneration systems, and reaction environments exhibits considerable universality. Over the years, the supplementation of core components and reaction conditions for eukaryotic CFPS has been meticulously evaluated.

### 5.1 DNA template

**Fig. 5:**
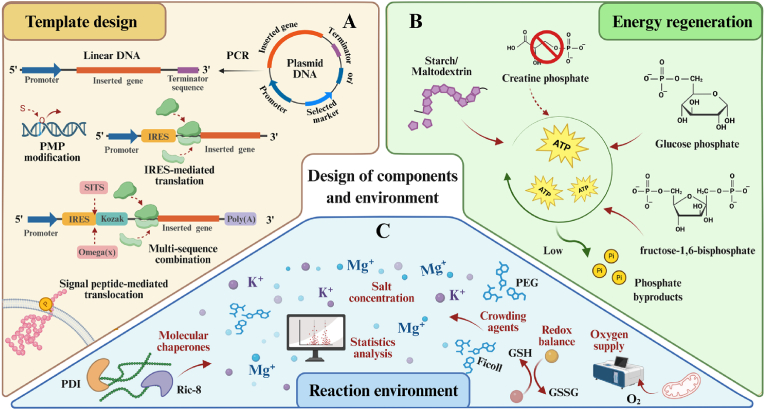
Optimization strategies for core components and reaction environments. (A) Engineering modifications to the template include linearization of the template structure, introduction of IRES elements and Kozak sequences, as well as SITS or enhanced Omega(X) sequences. These enhancements boost the translation efficiency of the eukaryotic CFPS system, and when combined with signal peptides, further facilitate protein transport. (B) Introducing sugar substrates, including glucose, fructose 1,6-bisphosphate, starch, or maltodextrin, to replace expensive high-energy phosphate compounds. (C) A standardized concentration screening protocol was established using statistical strategies to balance expression activity and ionic stability. Molecular crowding effects and redox environments were engineered, while oxygen supply was investigated to enhance reaction stability. Auxiliary systems such as molecular chaperones were incorporated to facilitate protein folding. Created with BioRender.com.

The core reaction of CFPS involves the transcription and translation of template DNA, making the rational design of templates crucial for the efficiency of eukaryotic CFPS systems (Fig. 5A). Innovations in template formats and engineered modifications to template sequences have significantly enhanced the translation efficiency of exogenous templates in eukaryotic CFPS systems. First, the template format was converted from plasmids to linear DNA, achieving high-throughput expression from DNA to protein. Integrating PCR template synthesis with the transcription-translation system significantly shortened the experimental cycle [101]. To address the susceptibility of linear DNA to degradation, phosphorothioate-modified primers (PMP) were employed to enhance the stability of PCR products [18]. Beyond template modification, alternative strategies include genomic modifications, the incorporation of nuclease inhibitors, and the addition of DNA-binding proteins. Through optimized design, the linear DNA templates generated by the two-step PCR method exhibit 60% higher performance than traditional plasmid templates [226]. Combined with expression vectors for large-scale protein production, this approach enables the parallel translation of 50 genes [12,13]. Second, the engineering modification of template sequences overcomes the bottleneck of inefficient eukaryotic translation initiation and endogenous suppression. By introducing viral-derived IRES elements, particularly those from *Cricket paralysis virus* (CrPV), *Encephalomyocarditis virus* (EMCV), and *Hepatitis C virus* (HCV), the cap-dependent translation initiation restriction was broken [44,222,227]. Integrating translation enhancer sequences, such as the 5′ untranslated region (UTR) from the *Ectropis obliqua nuclear* polyhedrosis virus gene, SITS, or the enhanced Omega (X) sequence, significantly boosts translation efficiency in eukaryotic CFPS systems [183,204,228]. Furthermore, employing multi-sequence combination strategies, such as integrating IRES elements with Kozak sequences, Poly(A) tails, or the influenza virus NS1 protein, significantly enhances target protein yields in eukaryotic CFPS systems [40,124,213]. To promote cotranslational translocation of complex proteins in the acellular system, the sequences of the honeybee melittin signal peptide and the α-mating factor prepro peptide were introduced [9]. Therefore, the rational design of composite template components significantly enhances the translational performance of the eukaryotic CFPS system.

### 5.2 Energy regeneration system

Protein synthesis requires substantial consumption of high-energy phosphate bonds, necessitating energy regeneration systems to sustain continuous extracellular protein expression. Traditional systems rely on high-energy phosphate compounds such as creatine phosphate and exogenous enzymes like creatine kinase, which are costly and produce phosphate byproducts that exert strong inhibitory effects [229]. Shifting from expensive energy substrates to alternative, cost-effective substrates is a core design strategy for the energy regeneration component of eukaryotic CFPS systems (Fig. 5B). The introduction of sugar substrates, including glucose phosphate, fructose-1,6-bisphosphate, or maltodextrin, provides energy through phosphorylation and glycolysis pathways, reducing the cost of cell-free reagents by 75% [9,[14], [15], [16]]. Hexametaphosphate and maltodextrin function as phosphate donors to achieve phosphate cycling [16,52]. The use of glycogen and soluble starch to slow glucose release extended the time of protein synthesis by 12 h [230]. The aforementioned energy regeneration design strategy enhances the cost-effectiveness of the eukaryotic CFPS system.

### 5.3 System environment

Beyond optimizing core components, the reaction environment of the CFPS system is also meticulously regulated (Fig. 5C). First, the preparation of cell extracts typically involves adding small amounts of salt supplements present in the buffers used for cell washing and resuspension prior to homogenization. The common buffer consists of HEPES-KOH buffer supplemented with 100 mM potassium acetate, 2 mM magnesium acetate, and 2 mM dithiothreitol [115,164]. HEPES buffer effectively maintains the CFPS reaction at a near-neutral pH environment. However, the ionic concentrations of each extract vary slightly. Magnesium and potassium ions are critical for influencing translation efficiency and stability. Multiple studies have established standardized concentration screening protocols to balance expression activity and ionic stability [19,40,110,214]. Supplementing polyamines at appropriate concentrations, particularly spermine and spermidine, can replace magnesium ions and increase peptide chain elongation rates [231,232]. Simultaneously, supplementation with metal cofactors such as iron ions and heme compensates for losses during extract purification [25]. However, the parameter evaluation of the CFPS reaction environment may depend on multiple variables. For complex components in the reaction mixture, design of experiments, deterministic screening design, or fed-batch supplement ratio processes simplify the procedure for exploring the effects of different variables [[17], [18], [19]]. Second, the synthesis of complex proteins requires the CFPS system to possess an appropriate redox environment and auxiliary folding systems. The addition of reduced and oxidized glutathione (GSSH/GSH), protein disulfide isomerase (PDI), disulfide bond C, or endoplasmic reticulum oxidoreductase enhanced product solubility and enabled co-translational folding of disulfide-linked proteins [141,151,168,233]. Supplementing molecular chaperones such as Ric-8 assisted in the correct folding of nascent polypeptide chains, thereby improving the yield of active complex proteins [234]. Next, to further simulate the intracellular environment, the effects of crowding and oxygen supply were investigated. Crowding agents such as PEG-8000, Ficoll-70, and Ficoll-400 induce molecular crowding that enhances transcription but may inhibit translation [164,235]. The segmented strategy, separating transcription (high crowding) from translation (low crowding), increased yield by 2.2 times. Furthermore, studies have revealed the critical role of oxygen supply in the synthesis of CFPS proteins in plants. Real-time monitoring and analysis using the μRAMOS-BioLector device, combined with respiratory chain inhibitors, confirmed the importance of mitochondrial function in maintaining the system's energy homeostasis [202]. These explorations provide robust support for optimizing eukaryotic cell-free reaction systems.

Overall, eukaryotic CFPS systems based on different extract chassis primarily focus on the rational design of core components, such as templates and energy regeneration systems, as well as on the regulation of reaction environments. As a vector for target protein expression, the primary development strategies for gene templates focus on high-throughput production, decapping independence, and component integration. These advancements have significantly streamlined preparation processes while comprehensively enhancing protein yield and template stability. Regarding supplementary components for the system, the energy regeneration system is central to sustaining the continuous operation of CFPS. By substituting expensive phosphate systems with inexpensive carbon sources and resolving feedback inhibition from metabolic byproducts, it balances cost and energy efficiency. Additionally, to maintain system stability, the reaction environment was finely tuned using statistical strategies. Simulating crowding effects, regulating redox potential, and supplementing auxiliary systems enhanced the eukaryotic CFPS system's adaptability for functional expression of complex proteins. In the future, the system's development should further adapt to diverse application scenarios, integrating machine learning algorithms to achieve high-throughput, automated expression.

## 6 Material integration of eukaryotic CFPS systems

The performance of eukaryotic CFPS systems depends not only on the optimization of biological components but also on the physicochemical properties of the reaction environment. The introduction of various advanced materials, such as porous framework materials, hydrogel matrices, and membrane simulants, not only promises to provide protective microenvironments for enzymes and membrane proteins, enabling reaction compartmentalization and functionalization, but also expands the applications of eukaryotic CFPS systems in fields such as biomanufacturing, biosensing, and *in vivo* therapy.

### 6.1 Enzyme encapsulation and protection in porous organic framework materials

**Fig. 6:**
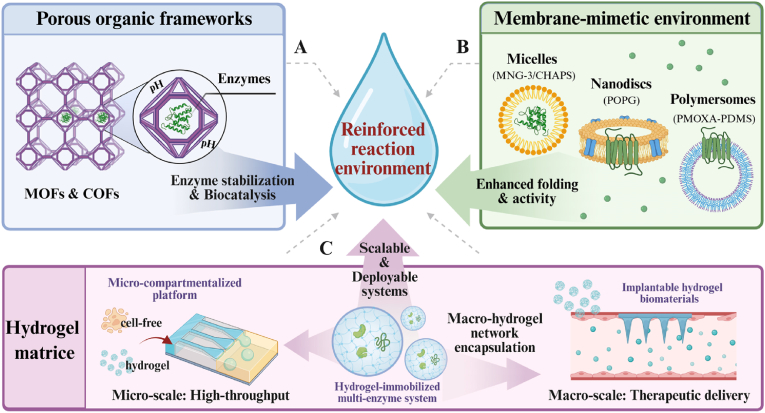
Integration of materials from frame encapsulation to membrane simulation environment construction. (A) The introduction of porous framework materials provides support for enzyme immobilization in eukaryotic CFPS systems and multi-enzyme cascade reactions. (B) Micelles formed by surfactants represent a classic strategy for membrane protein synthesis. Advanced mimetic materials, including nanodiscs and polymeric vesicles composed of amphiphilic block copolymers, have been introduced to provide enhanced mechanical stability. (C) The open porous structure of hydrogel materials provides physical support and a biocompatible environment for micro-compartmentalization within eukaryotic CFPS systems. Hydrogel-loaded cell-free platforms can be engineered as implantable materials. Created with BioRender.com.

Porous framework materials such as metal-organic frameworks (MOFs) and covalent organic frameworks (COFs) have been introduced for enzyme encapsulation and protection (Fig. 6A). These materials, with their highly tunable structures and excellent pore selectivity, can accommodate enzyme molecules of varying sizes by designing specific pore sizes and shapes. Surface modifications further enhance their interaction with enzymes, thereby improving enzyme activity and stability [236,237]. Meanwhile, MOF and COF frameworks effectively shield enzymes from harsh conditions like high temperatures, extreme pH values, and organic solvents, preventing enzyme leakage while enabling selective substrate diffusion [20]. This property holds immense potential for constructing highly efficient, stable, and recyclable biocatalysts, and offers novel carrier concepts for enzyme immobilization and multienzyme cascade reactions within eukaryotic CFPS systems.

### 6.2 Membrane-mimetic environment drive the synthesis of functional membrane proteins

The introduction of membrane-mimetic materials is crucial for meeting the requirements of eukaryotic CFPS systems for expressing membrane proteins (Fig. 6B). The functional expression of membrane proteins relies on hydrophobic environments, and surfactants represent a classic strategy to facilitate their soluble expression. Nonionic or zwitterionic surfactants, such as lauryl maltose neopentyl glycol (MNG-3), dodecyl octaethylene glycol ether, Fos-choline, and 3-[(3-cholamidopropyl)dimethylammonio]propanesulfonate (CHAPS), maintain the translational activity of eukaryotic CFPS systems while enabling efficient synthesis of transmembrane proteins like bacterial rhodopsin [[238], [239], [240]]. To more authentically mimic the natural lipid bilayer environment, membrane simulants such as liposomes, nanodiscs, exogenous microsomes, or inverted vesicles are widely employed [[23], [24], [25], [26], [27], [28], [29], [30]]. When combined with cell-free systems, they facilitate the co-translational insertion, purification, and structural characterization of membrane proteins [140,241,242]. As an emerging membrane simulation technique, the incorporation of POPG-based nanodiscs into the CFPS system has successfully enabled the functional synthesis of human endothelin receptor type B [243]. In addition to lipid structures, polymeric vesicles composed of amphiphilic block copolymers such as PMOXA-PDMS-PMOXAs have been introduced, exhibiting superior mechanical stability compared to liposomes [244]. These materials address the challenges of membrane protein aggregation and folding difficulties in CFPS systems by providing a hydrophobic environment or co-translational insertion mechanisms, holding promise for advancing the development of eukaryotic CFPS systems in biomanufacturing.

### 6.3 Reactive micro-compartmentalization and functionalization of hydrogel matrices

Hydrogel materials serve as excellent matrices at both macro- and micro-scales, providing the physical support and biocompatible environment required for eukaryotic CFPS systems (Fig. 6C). Various hydrogels, including agarose, sodium alginate, gelatin, and polyacrylamide, can support CFPS reactions [21,22]. Their high-water content and open pore structures facilitate reagent diffusion and protein synthesis. Especially at the macroscopic scale, hydrogel networks can act as structural macromolecular crowding agents, enabling highly efficient reactions without the need for additional reagents [21]. At the microscopic scale, micro-compartmentalization achieved through microchannels or microfluidic technology enables the construction of high-throughput, parallel-processable micro-reactors [245]. Furthermore, polyethylene glycol dipropionate-glycerol hydrogels can immobilize multi-enzyme systems, significantly extending enzyme half-lives and enhancing reusability [246]. Notably, by freeze-drying CFPS systems alongside template plasmids into agarose hydrogels, implantable materials are created that enable on-demand *in vivo* protein synthesis and delivery [10,39]. The research outcomes of these universal CFPS systems provide critical proof-of-concept validation and technical pathways for integrating materials into eukaryotic CFPS systems, enhancing their environmental adaptability and expanding their applications.

Material integration is pivotal to advancing eukaryotic CFPS development. Drawing on the successful experience of CFPS systems, porous frameworks, hydrogels, and membrane-mimetic materials offer novel approaches for constructing efficient synthetic platforms. However, significant challenges remain in their practical translation to eukaryotic systems. Enhancing material biocompatibility and encapsulation efficiency to accommodate eukaryotic components, overcoming diffusion limitations in hydrogels, addressing the difficulty of spontaneous insertion and lipid dependency of membrane proteins in artificial membranes such as liposomes and polymer vesicles, and balancing membrane stability with permeability are essential. Future research should focus on developing novel composite materials, elucidating the microscopic mechanisms of interaction between materials and biological components, and optimizing encapsulation processes to advance the large-scale application of eukaryotic CFPS in biopharmaceuticals, energy, and sensing applications.

## 7 Reactor design and equipment innovation for the eukaryotic CFPS system

Innovations in reaction modes and upgrades in equipment are key to enhancing the efficiency of eukaryotic CFPS, enabling large-scale production, and expanding its applications. Advances in reactor technology, from simple microbatch reactions to high-throughput microfluidic chips, have significantly broadened the application scenarios for eukaryotic CFPS.

### 7.1 Evolution of reaction modes from batch to continuous flow

**Fig. 7:**
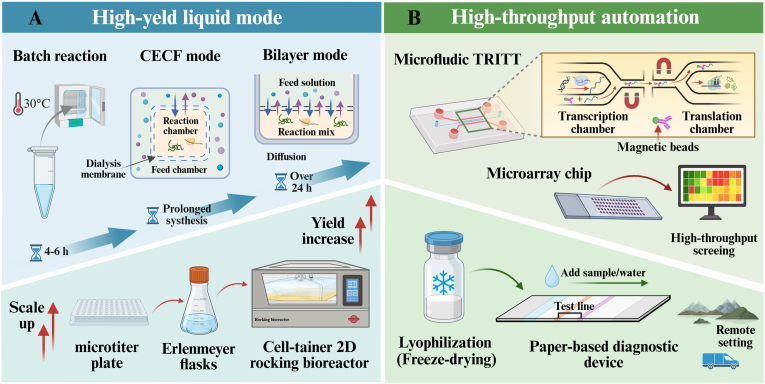
Evolution of reactor formats in eukaryotic CFPS system. (A) Multiple continuous and semi-continuous dialysis modes extend reaction times by supplementing substrates and removing byproducts. Shaker bioreactors scale up eukaryotic CFPS systems from 100-μL microplate formats by a factor of 20,000, enabling industrial-scale production. (B) The TRITT platform was developed based on magnetic particle-driven mRNA transfer between compartments. Microarray reaction modes enhance system throughput and shortens experimental cycles. Freeze-drying technology endows the eukaryotic CFPS system with deployment potential in harsh environments, while integration with paper-based materials facilitates the development of portable *in vitro* diagnostic devices. Created with BioRender.com.

Multiple continuous and semi-continuous reaction modes have been developed to overcome substrate depletion and byproduct accumulation in traditional batch reactions (Fig. 7A). CFPS reactions are typically conducted in batch mode in microplates or microcentrifuge tubes, with reaction volumes generally ranging from 10 to 50 μL. Compared to the batch mode, the semi-continuous reaction mode in the yeast CFPS system increased protein expression by more than 70% through substrate replenishment and byproduct removal [229]. By separating the reaction chamber from the feed chamber via a semipermeable membrane to continuously replenish substrates and remove byproducts, this dynamic equilibrium mode of continuous exchange cell-free (CECF) significantly extended reaction times to tens of hours, boosting protein yield from 40 μg/mL in batch reactions to approximately 1 mg/mL [42,135,206,247]. Particularly for energy systems relying on oxidative phosphorylation, CECF reactor designs effectively address oxygen transport limitations. To simplify dialysis apparatus operation, a “double-layer” mode was developed by placing the substrate buffer above the translation mixture [248]. This approach uses diffusion to deliver substrates slowly, facilitates automation, and sustains reactions for over 24 h. As the process matured, the eukaryotic CFPS reaction system was linearly scaled up from a 100 μL microtiter plate to a 1 L CELL-tainer® CT2D rocking-type bioreactor without loss of protein yield, achieving a 20,000-fold scale-up range [31]. The evolution of reaction patterns has maintained stable protein concentrations while expanding production scale, laying the foundation for industrial-scale manufacturing.

### 7.2 Equipment innovation and automation

To meet the demands of high-throughput screening, point-of-care testing, and automated synthesis, CFPS technology is advancing toward miniaturization, integration, and intelligence (Fig. 7B). A novel microfluidic platform achieves high automation for CFPS. This platform employs a compartmentalized design based on the “Transcription-RNA Trapping and Transfer-Translation” principle, utilizing magnetic particles to drive mRNA transfer between reaction chambers, effectively overcoming the mRNA degradation challenge inherent in standard CFPS systems [210]. This quasi-continuous operation mode supports the production of cytotoxic proteins and non-natural amino acid-labeled proteins. It can integrate microdialysis membranes and online monitoring sensors, providing new tools for automated drug synthesis. In terms of experimental automation design, customized Python scripts were developed to generate CSV command files that directly control the Echo acoustic liquid handler. This command set not only covers nanoliter-level precision liquid transfer steps and the automated preparation of standard curves, but also incorporates a key randomization feature for sample positions within 384-well plates [249]. This effectively eliminates positional effects—such as edge evaporation caused by pipetting time differences—and achieves full-process automation from experimental design to high-throughput sample preparation. Moreover, microarray reaction modes further enhance reaction throughput, enabling hundreds or thousands of independent reactions to occur simultaneously on the chip surface using minute amounts of reagents. This significantly accelerates the iteration speed of synthetic biology's “design-build-test” cycle [250]. The evolution of microfluidics and chip integration not only significantly enhances the automation level and screening throughput of eukaryotic CFPS systems but also lays a solid foundation for achieving portable field applications. To overcome reliance on cold-chain transportation and sophisticated laboratory equipment, the application of freeze-drying technology has endowed eukaryotic CFPS systems with remarkable portability, thereby expanding their potential for deployment in challenging environments. Through technical optimization, the developed freeze-dried version of the eukaryotic cell-free extract system maintains stability at room temperature for over two weeks, enabling the creation of “ready-to-use” reagents [111]. Indeed, freeze-drying CFPS systems onto paper-based materials offers an effective approach for constructing portable *in vitro* diagnostic devices and biosensors [251]. This strategy enables rapid pathogen detection, on-demand production of therapeutic proteins or antidotes in remote or harsh field conditions, advancing the development of on-site biomanufacturing.

Eukaryotic CFPS significantly enhances protein synthesis efficiency by scaling the continuous exchange reaction mode. Combined with microfluidic chips and freeze-drying technology, it achieves technological advancement from high-throughput automated screening to portable field applications. Nevertheless, current reaction equipment design still faces challenges, including insufficient functional integration of microfluidic systems, limited mass transfer efficiency during cross-scale amplification, and a lack of equipment adaptable to extreme environments, which constrain its stable operation in complex scenarios. Future efforts should focus on developing highly integrated reaction modules that combine dialysis and enhanced mass transfer functions. Integrating microelectronic sensors will enable precise online monitoring and regulation of reaction processes. Exploring hybrid reactor architectures to optimize system efficiency will further advance the deep application of high-end, intelligent reaction equipment in biomanufacturing and point-of-care diagnostics.

## 8 Applications of eukaryotic CFPS systems

The core value of the eukaryotic CFPS system lies in overcoming the limitations of traditional cell expression systems. It has become a universal tool for deciphering life mechanisms and constructing synthetic biology platforms, demonstrating broad application potential across multiple frontier fields. Through an open research environment, it not only provides effective solutions for deepening the understanding of fundamental life processes and developing artificial life systems but also significantly expands the boundaries of complex protein engineering, high-value-added compound development, and advanced biopharmaceutical research. This section primarily reviews the progress of the eukaryotic CFPS system in three key areas: elucidating fundamental life-science mechanisms, complex protein engineering applications, and metabolic engineering.

### 8.1 Fundamental research

**Fig. 8:**
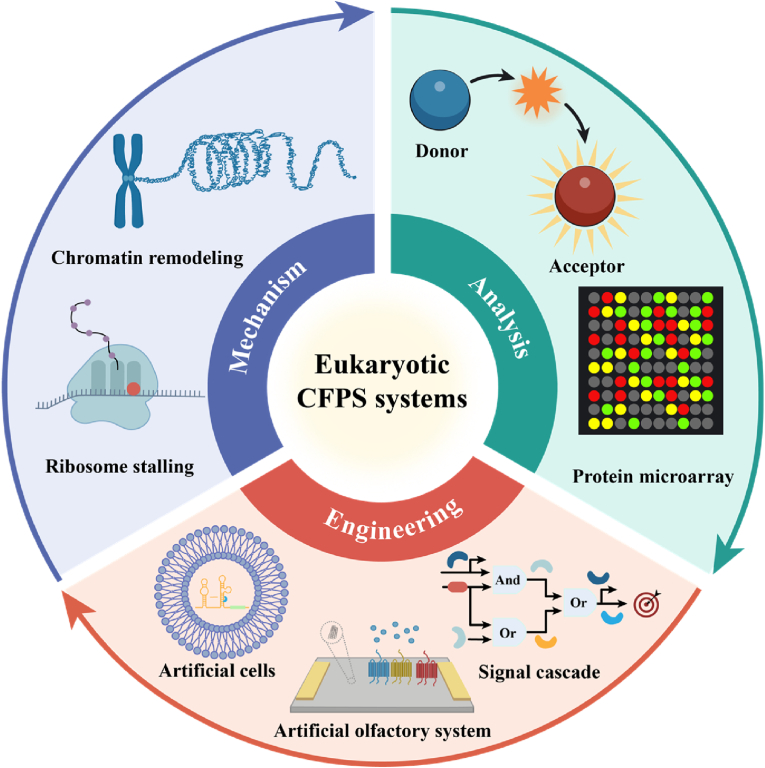
Eukaryotic CFPS systems for fundamental research. The eukaryotic CFPS system has deepened understanding of translation regulation mechanisms such as ribosomal termination. By reconfiguring chromatin, it provides an effective tool for cell-free genomics. High-throughput compound analysis is achieved through self-assembling protein microarrays combined with fluorescence amplification signals. Riboswitches developed based on ribosome blocking mechanisms provide an ideal synthetic biology platform for constructing programmed genetic circuits and artificial sensors through multi-step gene regulatory cascades. Combining with liposome encapsulation enables artificial cellular models for biosensors. Using microfabrication techniques, eukaryotic cell-free biosensor elements can be immobilized on electronic device surfaces to construct artificial olfactory systems.

Eukaryotic cell-free systems serve as effective tools for elucidating life mechanisms and constructing synthetic biology platforms. Facing challenges such as the complexity and difficulty of decoupling in traditional *in vivo* research environments, slow growth rates in living cell systems, and difficulties in genetic regulation engineering, the CFPS system offers a direct solution through its open environment and removal of growth constraints. It simplifies research models for chromatin and translational mechanisms, supports high-throughput protein structure analysis, and enables flexible integration of non-natural elements. This overcomes technical bottlenecks in living cells for gene circuit design, artificial cell construction, and bioelectronic interface development (Fig. 8).

The eukaryotic CFPS system, as an open and controllable reaction platform, has advanced the understanding of fundamental life mechanisms from the molecular to the supramolecular level. First, deciphering the eukaryotic translation mechanism is fundamental to optimizing heterologous protein expression and advancing drug development. Research on yeast extracts and RRL has provided deep insights into key aspects of eukaryotic translation, including the optimization of initiation, elongation, and termination elements [156,185,186,216]. Moreover, using this recombinant mammalian *in vitro* translation system, the mechanism by which eIF3j promotes the entry of release factors into the ribosome to regulate translation termination has been revealed, facilitating biochemical analysis of ribosomal translation regulation [160,218]. The yeast extract system reproduced the inhibitory effect of ribosome-stalling sequences on translation elongation, providing a streamlined model for studying translational regulation [176,177]. In-depth analysis of translation mechanisms enables the cell-free system to serve as an effective tool bridging fundamental molecular mechanisms and clinical pathology research. Research on ribosomal proteins such as RPSA, RPS3, and RPS6, as well as ribosomal inactivation proteins, has enhanced the understanding of the functions of disease-associated ribosomal proteins [163,217]. Evaluation of a tRNA YrdC-like family protein on KRAS gene translation using the RRL CFPS system revealed the crucial role of tRNA modification in regulating specific oncogene expression, thereby advancing understanding of the mechanisms underlying lenvatinib resistance [161]. Second, research into chromatin and transcriptional regulation offers new insights into the spatial specificity of gene expression. The DREX cell-free system successfully reconstructed complex chromatin containing physiological nucleosome spacing and non-histones, providing a realistic model for *in vitro* chromatin research [212]. In the reconstructed chromatin system, the intricate interactions among transcription factors such as CLAMP, MSL2, and GAF were thoroughly investigated, revealing the mechanism by which the dose compensation complex specifically targets the X chromosome [211]. By mimicking the competitive mechanisms of GAF and CLAMP to design artificially synthesized DNA-binding elements, it may be possible to anchor exogenous genes within specific chromatin environments, potentially enabling precise compartmentalized expression. The eukaryotic CFPS system serves as an effective tool for cell-free genomics, with promise to advance precision control in cell and gene therapy.

With a deepening understanding of life mechanisms, the eukaryotic CFPS platform has emerged as an effective tool for studying protein structure and function, supporting structural analysis techniques such as nuclear magnetic resonance, X-ray crystallography, and cryo-electron microscopy [138,194,252]. The multi-scale WGE-CFPS pipeline optimizes the structural biology research paradigm, enabling single-particle analysis under electron microscopy as well as two-dimensional and three-dimensional protein crystallography [253]. It provides a new platform for rapid production and purification of structural biology samples. Particularly for complex biomolecules, the eukaryotic CFPS system combined with fluorescent probe technology provides robust support for targeted protein assembly studies, such as viral proteins, membrane proteins, and their ligand complexes [138,184,194,206,209]. Moreover, mammalian extract systems demonstrate unique advantages in deciphering the oligomerization mechanisms and topological structures of key nuclear envelope and plasma membrane proteins. They provide a technical platform for elucidating the assembly principles of the linker of nucleoskeleton and cytoskeleton complex and resolving structural controversies surrounding membrane proteins [37,159]. Beyond structural analysis, eukaryotic CFPS systems combined with AlphaLISA or AlphaScreen technologies have enabled precise detection of protein interactions [110,112]. This method uses the formation of complexes between biotin- and FLAG-labeled proteins to trigger a chemiluminescent reaction upon proximity between donor and acceptor beads, enabling direct analysis of protein interactions in crude protein extracts without purification. Employing a self-assembled protein microarray format with fluorescence signal amplification, the eukaryotic cell-free protein interaction analysis system can screen 9600 compounds within 3 h, rapidly identifying potential activators or inhibitors and significantly shortening drug development cycles [133]. This eukaryotic cell-free high-throughput platform has been applied in large-scale serological screening and antibody detection, demonstrating promising clinical applications in disease biomarker discovery and vaccine candidate screening [197]. Leveraging their rapidity, high throughput, and miniaturizability, eukaryotic cell-free systems play a vital role in proteomics and high-throughput screening.

The eukaryotic CFPS system overcomes the limitations of live cell growth and the complexity of the eukaryotic intracellular environment, providing an ideal synthetic biology platform for constructing programmable genetic circuits and artificial sensors. Traditional biological detection techniques typically rely on sophisticated instruments and complex sample pretreatment. Research has identified a ligand-dose-dependent *cis*-regulatory RNA element in the mRNA 5′ UTR that blocks ribosome loading or mRNA scanning, enabling sensing and response to various metabolites [[254], [255], [256], [257]]. Through diverse aptamer designs, such as single aptamers, multiple aptamer copies, or split aptamers, precise translation regulation can be achieved in WGE or RRL systems [[258], [259], [260]]. Leveraging this ribosome-blocking mechanism, eukaryotic cell-free systems have been successfully employed to develop protein-responsive upregulating riboswitches (ON-riboswitches), offering an alternative for immediate *in vitro* detection. To enhance signal intensity, the study introduced an aptazyme gene regulation strategy. By fusing the aptazyme to the 5′-UTR of the luciferase gene and utilizing a cofactor-induced self-cleavage mechanism to release mRNA, detection sensitivity was significantly increased 20-fold, outperforming sensors reliant on prokaryotic translation systems [261,262]. Furthermore, rational design of eukaryotic pt-ON-riboswitches based on IRES has further enhanced the versatility of eukaryotic cell-free biosensors. By introducing sequences for anti-IRES, aptamers, anti-anti-IRES, and modulators, highly specific riboswitches for theophylline, FMN, tetracycline, and sulforhodamine B have been constructed [263,264]. Simultaneously, through the exploration of multiple regulatory mechanisms, including ribosome shunting, aptamer-regulated sup-tRNA processing, and translation modulation by barley yellow dwarf virus 3′ cap-independent translation element, a rich array of components has been provided for synthetic biology, supporting precise control of gene expression and the construction of complex genetic circuits [[265], [266], [267]]. Furthermore, it is feasible to construct highly complex, programmable eukaryotic gene circuits using cell-free systems. A novel ON-riboswitch optimized based on hammerhead ribonuclease (HHR) achieved a 20-fold increase in expression levels by leveraging HHR's self-cleavage mechanism [257]. Building on this, a multi-step gene-regulation cascade was constructed by integrating similar small-molecule-responsive ON-riboswitches, enabling highly orthogonal control over multiple gene circuits [32]. These diverse translational regulatory strategies and complex gene cascades lay a solid foundation for the development of highly sensitive *in vitro* biosensors and complex programmable genetic networks.

In addition to RNA-based response elements, eukaryotic CFPS systems offer unique advantages in the rapid prototyping of gene regulation modules. Using HeLa cell extracts and automated acoustic liquid-handling technology, this platform not only enables high-throughput parallel screening of T7 promoter variants and EMCV IRES variants but also accurately reproduces complex regulatory dynamics—such as CRISPR/dCas9 transcriptional repression and L7Ae translational repression—in a cell-free environment [249]. Crucially, the quantitative characteristics of key regulatory modules exhibit high fidelity to live-cell data. This strategy effectively overcomes the limitations of traditional mammalian cell culture—namely, slow growth and difficulty in high-throughput characterization—providing an efficient *in vitro* iterative testing platform for the optimization of genetic modules. Currently, this automated prototyping platform has been successfully extended to the field of plant synthetic biology. Leveraging a highly automated chloroplast-based cell-free gene expression workflow, the study enabled rapid prototyping and systematic characterization of 38 different regulatory elements, including 5′UTR, 3′UTR, and endogenous chloroplast promoters, across evolutionarily diverse species such as spinach, wheat, and poplar [98]. This approach not only bypasses time-consuming *in vivo* cloning and screening cycles but also confirms the cross-species compatibility of genetic elements, providing an efficient *in vitro* iterative testing platform for breeding stress-tolerant crops and engineered trees. In short, this automated *in vitro* prototyping platform overcomes the limitations of *in vivo* testing, paving the way for standardized, high-throughput design in eukaryotic synthetic biology.

Eukaryotic cell-free systems demonstrate significant application value in the fields of artificial cell construction and bioelectronic interfaces. By co-encapsulating CFPS systems with DNA encoding various genes within cell-sized compartments, artificial systems mimicking cellular functions can be constructed. Therefore, integrating liposome encapsulation technology, the WGE CFPS system employed an artificial cell model to construct three eukaryotic artificial cell biosensors [193,198]. With their independent, stable responsiveness, these artificial cells enabled simultaneous detection of multiple analytes, with broad linear response ranges and high sensitivity. Meanwhile, regulating the release of signaling molecules from artificial cells via riboswitches can expand the application of cell-free systems in biosensing and intercellular communication [33]. Additionally, in the field of olfactory sensing, a novel odor sensor was developed by integrating eukaryotic cell-free-synthesized olfactory receptors with graphene field-effect transistors [250]. The bioelectronic interface created by immobilizing highly specific biomolecular recognition elements onto the surface of electronic devices not only exhibits specific responses to ligands at varying concentrations but also demonstrates high sensitivity and a wide dynamic range. By integrating transistor arrays onto chips using microfabrication techniques, this platform holds promise for scaling to hundreds of olfactory receptors, enabling the construction of artificial olfactory systems capable of recognizing complex odors. With the advancement of synthetic biology technologies, these finely tuned regulatory mechanisms are expanding into broader practical applications through the construction of novel artificial cells and bioelectronic interfaces.

Accordingly, the eukaryotic CFPS system has made progress in both fundamental research and applied translation. Molecular mechanisms such as translation initiation and chromatin competition have been elucidated, and precise gene expression control has been achieved using aptase enzymes, IRES riboswitches, and multi-step cascade circuits. By integrating liposome encapsulation with graphene transistor technology, this system has further enabled the construction of high-performance artificial cellular sensors and olfactory electronic interfaces. However, practical challenges remain, including insufficient sensor stability, unvalidated signal-to-noise ratios and detection limits, and the absence of standardized operating procedures [255]. Future research should focus on optimizing component performance through computational design and high-throughput screening, establishing standardized protocols to enhance system robustness and meet practical application demands.

### 8.2 Protein engineering

**Fig. 9:**
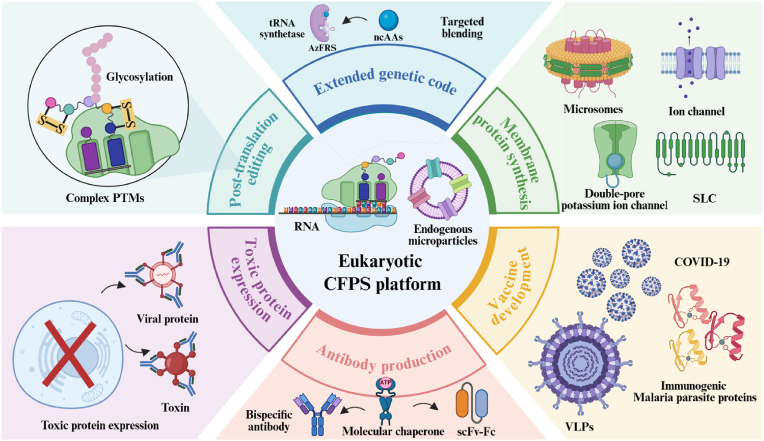
Eukaryotic CFPS systems for protein engineering. This figure demonstrates the comprehensive capabilities of the eukaryotic CFPS system in protein engineering, spanning from tool development to therapeutic applications. The incorporation of ncAAs and the expression of toxic proteins demonstrate the system's openness and tolerance. Leveraging the synthesis of diverse membrane proteins enhances the system's folding and transmembrane capabilities, while PTMs enable chemical functional regulation. This supports the structural assembly of vaccine proteins and the integration of therapeutic antibodies, reflecting the eukaryotic CFPS system's progression from simple biomanufacturing to complex, high-end drug applications. Created with BioRender.com.

The eukaryotic CFPS system, with its rapid expression capability and endogenous microsomal structure, has become a vital tool for fundamental research and engineering applications involving complex proteins. Addressing common challenges in protein synthesis, such as folding and modification difficulties alongside production cost constraints, this system offers effective solutions through its openness and flexibility. By overcoming genetic code constraints, circumventing cytotoxicity, integrating endogenous membrane environments, and regulating PTMs, the eukaryotic CFPS system has successfully established an application chain spanning the synthesis of difficult-to-express proteins, the assembly of complex vaccine antigens, and the development of high-end antibody drugs (Fig. 9).

Site-directed incorporation of non-canonical amino acids (ncAAs) overcomes the limitations of traditional genetic coding with 20 amino acids, endowing proteins with novel physicochemical properties and biological functions. Within cells, amino acid attachment to tRNA requires catalysis by aminoacyl-tRNA synthetases (aaRSs). However, challenges such as membrane permeability, metabolic burden, and interference from orthogonal systems make flexible incorporation of multiple ncAAs difficult to achieve. In contrast, eukaryotic CFPS systems have undergone substantial iterative updates and optimization for genetic code expansion. These platforms facilitate site-specific ncAA incorporation either by chemically charging adapter tRNAs that decode four-base or amber codons, or by employing engineered aaRS/tRNA pairs for orthogonal acylation [[268], [269], [270]]. Given this, the glutamyl-tRNA synthetase pair and tyrosyl-tRNA synthetase (TyrRS) pair exhibit excellent orthogonality in eukaryotic translation systems, making them candidate tools for specific incorporation of ncAAs [271,272]. Engineered TyrRS variants, specifically Azido-L-phenylalanyl-tRNA synthetase, have been demonstrated to achieve targeted incorporation of azido-l-phenylalanine in eukaryotic CFPS systems, with rational optimization of the amber suppressor tRNA significantly boosting incorporation efficiency [206,273]. Additionally, the engineered pyrroline lysine system exhibits inherent orthogonality and broad substrate specificity, significantly expanding the range of ncAAs that can be incorporated [274,275]. However, this strategy of exogenously adding aaRS/tRNA pairs is often accompanied by complex purification processes that limit cell-free genetic code expansion. To streamline the orthogonal translation system (OTS) construction process, a universal expression platform capable of producing endogenous orthoenzyme extracts was engineered by genetically modifying host cells [165,276,277]. This endogenous genetic expansion strategy lays a solid foundation for the functional regulation of complex proteins and the development of high-end pharmaceuticals. The eukaryotic cell-free OTS enables the introduction of minute probe molecules without disrupting protein conformation, overcoming the issue of traditional large-molecule fluorescent tags such as GFP interfering with protein folding and function [278]. Based on photoaffinity cross-linking and azide-based chemically selective fluorescent labeling techniques, the dynamic conformation and integrity of proteins have been elucidated [206,207,279,280]. Direct incorporation of ncAAs bearing glycosylation structures, such as glycosylated serine and tyrosine derivatives, enables monosaccharide or disaccharide modifications at specific sites [281]. By introducing these ncAAs with unique chemical properties into eukaryotic cell-free OTS systems, functions such as site-specific protein conjugation and long-lasting protein modification have been achieved. This approach holds promise for expanding the engineering boundaries of therapeutic proteins, including antimicrobial peptides and antibody-drug conjugates [[282], [283], [284]].

Toxic proteins, particularly viral proteins, hold significant value for understanding viral life cycles, infection mechanisms, and the development of antiviral drugs. CFPS technology effectively circumvents biosafety restrictions and host cell toxicity by using crude cell lysates rather than live cells. It enables the direct synthesis of functional toxic proteins without complex purification, making it a rapid response tool for addressing emerging public health emergencies such as COVID-19. Leveraging the key advantages of an open environment, the eukaryotic CFPS platform has successfully synthesized multiple infectious viruses or their critical components, including picornaviruses, EMCV, poliovirus, HCV, and rift valley fever virus [18,62,138,189,227,[285], [286], [287], [288], [289], [290], [291], [292]]. This has effectively supported in-depth analysis of viral protein properties and processing mechanisms, revealing the processing mechanism of EMCV 2A-2B, the role of membrane structures in HCV protein maturation and co-translational cleavage of HCV polyproteins, and the critical value of the 14S subunit as a key intermediate in poliovirus RNA encapsulation during poliovirus cell-free synthesis [[293], [294], [295]]. Additionally, this system is not only suitable for the rapid expression and characterization of toxic proteins such as coat proteins, envelope proteins, and bacterial toxins, but also serves the development of antiviral drugs that inhibit viral replication or block viral protein functions, as well as vaccine design [93,170,220]. *In vitro* translation of viral genes, such as hepatitis E virus capsid protein, non-structural proteins, structural proteins, and accessory proteins derived from SARS-CoV-2, along with various Shiga toxin variants, has significantly advanced drug target discovery for combating large-scale infectious diseases [169,170,172]. The eukaryotic cell-free system has demonstrated the potential to synthesize and characterize complex virulence proteins, which are crucial for antiviral drug development and screening.

Membrane proteins are central to biological functions and drug development [6,296]. Their hydrophobicity and structural complexity pose barriers for traditional preparation methods, which often fail to yield correctly folded proteins or result in loss of functional activity [297,298]. The eukaryotic CFPS system, with its open environment and membrane-mimicking capabilities, serves as a flexible platform for membrane protein expression. It provides robust support for fundamental research and drug development targeting key molecules such as ion channels, transporters, and G-protein coupled receptors (GPCRs). The eukaryotic CFPS system has successfully synthesized multiple channel proteins within endogenous membrane environments such as microsomes, including key cardiac transmembrane channels hERG and hNaV1.5, the potassium channel KcSA, the ligand-gated ion channel nAChR,and over 95% of up to 250 human channel proteins—most of which retain voltage-dependent opening properties [28,35,45,[299], [300], [301]]. Additionally, this system has demonstrated promising performance in investigating complex assembly mechanisms, particularly in elucidating the heterodimer formation between TREK-2 and TWIK-1 in dual-pore potassium channels [302]. This provides novel approaches for understanding pain pathophysiology and drug screening. Beyond channel proteins, the solute carrier family is the largest class of transporters, facilitating the transmembrane transport of various substances and representing a highly promising drug target [303]. Addressing the challenges of structural complexity and poor stability in living cells, the eukaryotic CFPS system has successfully expressed 22 human SLC transporters from 20 distinct families and synthesized complex human serotonin transporter and sarco/endoplasmic reticulum Ca2+/ATPase pumps [34,35]. Finally, GPCRs constitute the largest family of membrane receptors in the human body, regulating the vast majority of cellular signaling pathways, yet their structure and function remain largely unexplored. The eukaryotic CFPS technology overcomes the challenges of low GPCR expression levels and susceptibility to inactivation in traditional systems, enabling the production of multiple GPCRs and direct analysis of their ligand-binding properties [209,304]. Notably, it has successfully synthesized the gamma-aminobutyric Acid B Receptor and demonstrated the correct assembly of its heterodimers [305]. With the continuous advancement of analogues, eukaryotic CFPS technology offers a promising approach for drug screening and research targeting mitochondria.

PTMs of proteins significantly expand the complexity and functional diversity of the proteome, playing an irreplaceable role in elucidating disease mechanisms and drug development [306,307]. Nevertheless, the functional complexity and highly dynamic nature of proteins make the study of PTM challenging. The eukaryotic CFPS system, leveraging its open environment and precise control over reaction conditions, successfully mimics multiple critical modifications, including glycosylation, phosphorylation, lipidation, acylation, ubiquitination, and prenylation. This provides an efficient platform for preparing complex therapeutic proteins and developing functional enzymes. First, the functional activity of therapeutic proteins such as antibodies and hormones highly depends on proper glycosylation modifications [308]. The eukaryotic CFPS system has successfully achieved efficient synthesis of functional glycoproteins by integrating endogenous or exogenous microsomes [18,93,96,146,167,[309], [310], [311], [312]]. It demonstrates precise control over N-linked glycoforms, particularly in the production of complex hormones such as erythropoietin, overcoming the significant batch-to-batch variability inherent in traditional methods [118,313]. In addition to glycosylation, modifications such as phosphorylation, lipidation, and disulfide bond formation are equally crucial for the proper folding and functional activation of proteins [43,157,280,[313], [314], [315], [316], [317]]. The CFPS system, based on eukaryotic extracts from insects and plants, achieves simulation of multiple modifications including N-myristoylation, N-acetylation, and phosphorylation [[58], [59], [60], [61]]. Furthermore, the microsomal lumen is more favorable for the synthesis of proteins with disulfide bonding activity [205,318]. By optimizing the redox environment and adding folding enzymes, it promotes the generation of complex proteins with active disulfide bond structures [151,168,233,234]. However, PTM research based on eukaryotic CFPS systems remains focused on common types. New research highlights the impact of post-translational modifications such as lactonylation and succinylation on the tumor microenvironment, suggesting they may play a significant role in immunotherapy [319]. Eukaryotic cell-free platforms require continuous expansion to support research on these novel PTMs, thereby enhancing the understanding of their role in disease mechanisms and providing new insights for drug development and screening.

Vaccines are a key tool for preventing human diseases. Common vaccine design strategies include live attenuated, inactivated, subunit, conjugate, and virus-like particles (VLPs), but these approaches are constrained by challenges such as difficulties in recombinant protein expression and insufficient screening of critical vaccine targets [320]. The eukaryotic CFPS platform is an optimized choice for producing high-quality complex vaccine antigens and toxoids, meeting the demands for rapid, cost-effective, and cytotoxic-resistant vaccine synthesis [224,321]. Malaria caused by *Plasmodium falciparum* is a major disease affecting global public health security, with nearly half of the world's population at risk of infection [322]. Given the current lack of understanding regarding protective immunity targets against malaria and the absence of effective vaccines, the WGE CFPS platform was used to produce immunogenic malaria parasite proteins at high throughput [311,[323], [324], [325]]. Combined with a screening system, this approach successfully identified over 100 potential protein targets and revealed the developmental patterns of immunity to malaria in children, providing robust support for malaria vaccine design [197,326]. Moreover, the eukaryotic CFPS platform's capability for rapid synthesis of key viral proteins has also been applied to COVID-19 vaccine research, enabling the synthesis of the complete RBD critical region of SARS-CoV-2 and high-throughput screening of compounds that inhibit RBD-ACE2 interactions [141]. Beyond traditional vaccine candidate screening, VLPs mimic the structural conformation of natural viruses and are widely used as nanoparticle carriers in vaccines, drug delivery, and gene therapy [[327], [328], [329], [330]]. Addressing the challenges of poor stability and difficult purification in most *in vivo* VLP expression systems, the eukaryotic CFPS system enables efficient, stable synthesis and scalable production of various viral VLPs, including human papillomavirus 58 L1 capsid protein, hepatitis B virus core protein, and HIV immature capsid [157,188,189,203,220,331]. Eukaryotic CFPS systems represent a promising option for producing vaccine candidates, accelerating vaccine design and development.

Antibodies are a vital component of the immune system, capable of recognizing and neutralizing pathogens. However, high production costs and complex PTM requirements have limited their ability to conduct high-throughput screening and scale up production. The eukaryotic CFPS system simplifies the technical requirements for complex antibody production, enabling large-scale screening, rapid synthesis, and precise diagnostics. By supplementing protein disulfide isomerase, molecular chaperones, and redox systems, it enables the synthesis of antibody fragments with correct solubility and functionality, such as immunoglobulin G and single-chain variable fragments (scFv) Fc fusion proteins [148,149,171,233,332]. Synthetic biotinylated scFvs can be immobilized on streptavidin-coated solid surfaces to bind antigens, while incorporation of non-canonical amino acids enables co-translational fluorescent labeling of the antibody fragments [149]. Furthermore, bispecific antibodies leverage their unique dual-target recognition capability to significantly enhance therapeutic efficacy through synergistic effects. By employing the “knobs-into-holes” strategy in conjunction with the CHO CFPS platform, efficient *in vitro* assembly and production of bispecific antibodies have been achieved, offering a novel solution pathway for treating multiple targets [225]. Building on this foundation, specific antibodies targeting the Middle East respiratory syndrome coronavirus and the Trichodysplasia spinulosa-associated polyomavirus were expressed on the WGE CFPS platform [332,333]. In the field of *in vitro* diagnostics, this system constructed an A-cube high-throughput antibody chip by synthesizing eight key antigens and immobilizing them on solid-phase carriers, enabling auxiliary diagnosis of autoimmune connective tissue diseases [196]. This novel A-cube autoantibody detection method ensures testing efficacy while avoiding the radioactive risks associated with traditional immunoprecipitation methods, providing a powerful tool for comprehensive analysis of autoantibody profiles. The eukaryotic CFPS platform has broad application prospects in precise typing and personalized therapy.

The eukaryotic CFPS system has made remarkable progress in protein engineering. Leveraging its ability to overcome cell membrane limitations and tolerate toxic reactions, it has successfully enabled the efficient synthesis of complex toxic proteins, membrane proteins, antibodies, and VLPs. By integrating ncAAs with PTM technologies, it has significantly expanded the functional and application boundaries of proteins. However, the technology currently faces challenges, including insufficient incorporation efficiency of multiple ncAAs, difficulty in completely eliminating endogenous competition, room for improvement in protein yield, and high costs for large-scale production. Future research will focus on optimizing reaction systems through genome engineering, tRNA manipulation, and novel translational factor development. Concurrent efforts will prioritize cost reduction while integrating cutting-edge technologies, including artificial intelligence, high-throughput screening, and continuous-flow reactions. This will support novel modification studies, complex glycoprotein expression, and the development of diversified drug delivery systems, thereby driving comprehensive breakthroughs in antibody engineering, vaccine development, and innovative drug screening.

### 8.3 Metabolic engineering

Traditional microbial cell factories are often constrained by the complex metabolic network regulation of the host when constructing intricate metabolic pathways, resulting in prolonged pathway characterization cycles and poor adaptability. Simultaneously, plant-derived secondary metabolites frequently exhibit low yields in living cells due to toxicity or pathway competition. In contrast, eukaryotic CFPS technology creates an open, controllable *in vitro* synthetic environment that effectively removes constraints on cellular growth. This approach not only addresses issues of product toxicity and metabolic competition but also provides a crucial platform for pathway analysis, the discovery of novel catalytic elements, and the efficient synthesis of high-value-added metabolites.

**Fig. 10:**
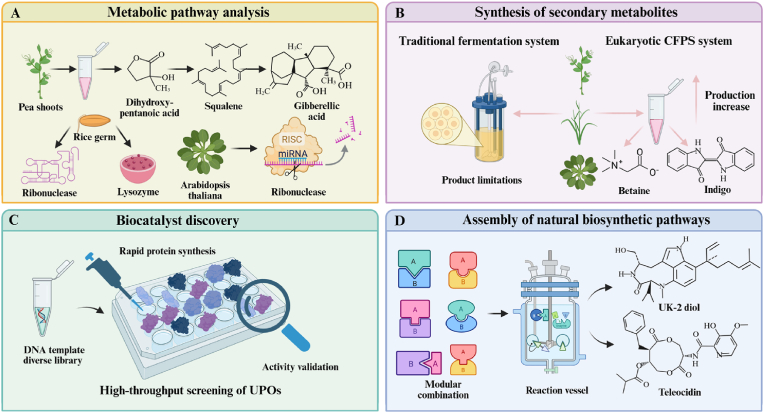
Eukaryotic CFPS systems for metabolic engineering. (A) Plant CFPS systems, such as those in peas, Arabidopsis, and rice, have achieved the conversion of squalene and the biosynthesis of gibberellin and multiple enzymes, demonstrating their potential for metabolic pathway analysis and enzyme studies. (B) The synthesis of various plant-derived secondary metabolites, such as indigo and betaine, demonstrates the superior performance of eukaryotic CFPS systems over traditional fermentation in this application scenario. (C) For key components exhibiting high catalytic diversity, this system enables the expression and screening of multiple UPOs. (D) The eukaryotic CFPS system enables rapid metabolic pathway reconfiguration through the modular assembly of diverse enzymes. Created with BioRender.com.

The eukaryotic CFPS system is suitable for metabolic pathway analysis and enzyme mechanism research (Fig. 10A). Addressing the challenges of complex plant metabolic pathways and the difficulty of precisely analyzing post-transcriptional regulatory mechanisms *in vivo*, a plant-derived cell-free system based on peas has successfully achieved the conversion from 2-(14) C-mevalonate to squalene and the biosynthesis of gibberellin [77,81,334]. This provides an effective tool for *in vitro* reconstruction and analysis of complex plant metabolic pathways. The *A*. *thaliana* system is specifically used to decipher post-transcriptional regulatory mechanisms in plants [83]. Concurrently, the rice embryo system has demonstrated the potential of plant systems for studying enzyme characteristics and developing enzyme preparations *in vitro*, successfully synthesizing biologically active ribonuclease and lysozyme [143]. However, the yield of plant-derived secondary metabolites is limited in traditional fermentation systems. The eukaryotic CFPS system achieves efficient synthesis of compounds (Fig. 10B) such as betanin, lycopene, indigoidine, betanin, and betaxanthin by reducing side reaction competition and central metabolic consumption, with some products yielding over 30 times higher than in living cell systems [200]. CRISPR-optimized yeast-based cell-free factories also enable high-rate production of 1,4-butanediol [187]. Non-specific peroxidases (UPOs), as key components with high catalytic diversity, are abundant in natural sources yet difficult to express heterologously. The eukaryotic CFPS system supports the rapid synthesis and activity assessment of diverse UPOs (Fig. 10C), such as MroUPO, AaeUPO, and PanUPO, effectively overcoming host expression barriers [131,151]. Notably, the eukaryotic CFPS system also excels at reconstructing complex natural product biosynthetic pathways (Fig. 10D). Using the BYE cell-free system, the teleocidin biosynthetic pathway was reconstructed, and with cofactor supplementation, the yield of teleocidin B-3 exceeded 80 mg/L. In the WGE CFPS system, a metabolic pathway comprising 10 proteins was reconstructed, achieving the first-ever cell-free biosynthesis of commercially valuable UK-2 diol [201]. This demonstrates the system's unique advantage in producing high-value-added complex natural products. Upon this foundation, the modular assembly of metabolic pathways based on the eukaryotic CFPS system avoids the cumbersome steps and lengthy optimization cycles required to construct cross-species pathways within cells. By engineering lysates of different enzymes into “plug-and-play” modules, the modular assembly and rapid reconfiguration of metabolic pathways are achieved through mixing lysates from diverse sources, significantly shortening the “design-build-test” cycle [36]. The developed hybrid modular platform has been applied to the prototypical synthesis of compounds such as borneol and menthol.

The eukaryotic CFPS system not only overcomes the limitations of traditional cell factories in product synthesis and pathway construction but also accelerates iterative optimization in metabolic engineering through modular assembly and catalyst discovery, demonstrating immense application potential. However, in long-cycle, multi-step cascade reactions, the lack of compartmentalization by cell membranes for metabolic intermediates can allow cytotoxic intermediates and byproducts to directly inhibit key enzyme activities in open *in vitro* systems. Furthermore, the high cost of reaction system preparation and the absence of standardized processes for large-scale amplification hinder the transition from laboratory-scale micro-production to industrial-scale manufacturing. Looking ahead, integrating low-cost continuous-flow processes with AI-assisted enzyme engineering design will propel eukaryotic cell-free systems toward greater efficiency and standardization. This evolution holds promise for overcoming laboratory constraints and realizing true industrial-scale biological manufacturing.

## 9 Challenges

Despite significant strides in optimizing eukaryotic cell-free protein synthesis systems over the past few decades, their translation from niche laboratory tools to universal production platforms remains hindered by systemic bottlenecks. These challenges are not merely technical but foundational, spanning economic feasibility, biological fidelity, operational scalability, and theoretical depth. Specifically, the field currently faces four intertwined obstacles: the persistent trade-off between production yield and cost, the difficulty in replicating complex post-translational modifications, the lack of standardization required for automation, and the constraints imposed by an insufficient mechanistic understanding.

### 9.1 The challenge of balancing output and costs

**Fig. 11:**
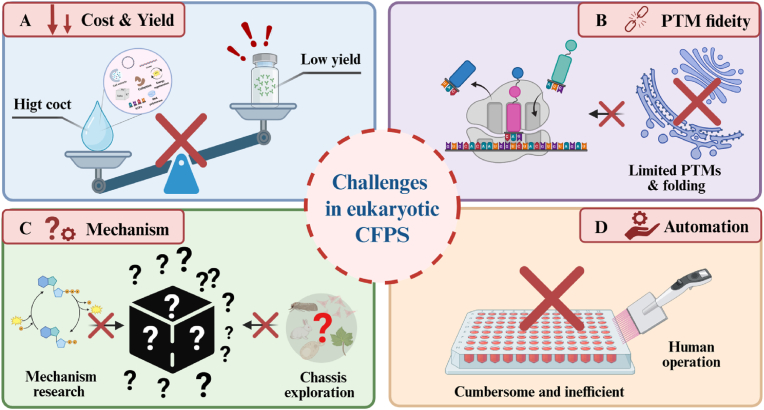
Challenges of eukaryotic CFPS system. (A) The high cost of eukaryotic CFPS systems, stemming from their dependence on expensive energy substrates and supplemental reagents, creates a significant imbalance with their low efficiency in biomanufacturing applications such as vaccines and antibodies. (B) Certain eukaryotic extracts lack endoplasmic reticulum-derived vesicular structures. Protein folding relies on random incorporation into artificial membranes rather than directed assembly, thereby limiting complex PTMs of functional proteins. (C) The relationship between novel chassis characteristics and system performance remains unclear. The “black-box” limitations of eukaryotic extract metabolism and regulatory network mechanisms constrain system development. (D) The limited versatility of eukaryotic CFPS systems prevents the implementation of simplified high-throughput and automated workflows. Created with BioRender.com.

The imbalance between cost and yield is an inherent issue in eukaryotic CFPS systems (Fig. 11A). Compared to highly optimized prokaryotic systems, eukaryotic CFPS systems generally exhibit lower protein yields. Although strategies like continuous exchange have improved productivity, the cost-effectiveness of systems such as wheat germ extract remains inferior to established bacterial platforms and industrial fermentation. A primary bottleneck in the extraction process is its labor-intensive, inefficient nature, which typically recovers only milliliter volumes of active material from large quantities of substrate. This issue is compounded by biological variability in raw materials, which precludes the consistency required for standardized manufacturing. Furthermore, the reliance on expensive energy substrates and supplementation reagents exacerbates operational costs. Consequently, the imbalance between high construction costs and limited protein yields remains a central obstacle to scaled-up production.

### 9.2 Precise regulation of functional protein PTMs

Although plant extracts possess basic eukaryotic modification capabilities, the types, efficiency, and fidelity still fall short of those of natural mammalian cells (Fig. 11B). Fundamental limitations persist for proteins that require complex post-translational modifications. The standard reducing environment hinders disulfide bond formation, and species-specific differences in glycosylation pathways may lead to the production of immunogenic compounds. Current remedial strategies involving the addition of exogenous folding factors are often patchwork solutions that suppress translation efficiency without achieving precise endogenous regulation. Furthermore, membrane protein assembly is highly inefficient due to the absence of endoplasmic reticulum-derived microsomes, which prevents the use of translocation complexes for co-translational insertion. Consequently, folding relies on stochastic integration into artificial membranes rather than directed assembly, resulting in low functional recovery and restricting utility for high-value biologics.

### 9.3 Chassis exploration and mechanism research

The advancement of eukaryotic CFPS is constrained by insufficient mechanistic understanding and a scarcity of available chassis (Fig. 11C). At the mechanistic level, core characteristics of energy metabolism and regulatory networks remain largely opaque. Key dynamics such as ATP equilibrium and waste accumulation are not well quantified, forcing optimization strategies to rely on phenotypic trial-and-error rather than rational design based on metabolic models. Concurrently, existing chassis suffer from intrinsic limitations in either preparation difficulty or modification capacity. A systematic understanding of the structure-function relationship between chassis properties and system performance is still elusive. There is an urgent need to explore novel chassis and conduct in-depth mechanistic studies to overcome these performance ceilings.

### 9.4 Insufficient system standardization and automation

Eukaryotic systems lag significantly behind prokaryotic platforms regarding standardization and process automation (Fig. 11D). This limitation stems primarily from complex preparation procedures that are difficult to standardize. Extracts like wheat germ require intricate processing, during which strict physiological control of the raw material is essential, making it difficult to establish reproducible standard operating procedures. On the application side, significant variations in expression levels among different proteins necessitate bespoke purification and quantification steps, preventing the implementation of streamlined, high-throughput workflows. Moreover, the complex reaction formats often required to boost yields are incompatible with miniaturized automation. The absence of a fully automated platform connecting DNA templates to functional data remains a critical technical bottleneck.

## 10 Prospects

While eukaryotic CFPS systems have demonstrated unique advantages in expressing complex proteins, their transformation into universal biomanufacturing platforms is currently hindered by the systemic challenges outlined previously. Moving forward, the field is expected to transition from empirical optimization to rational engineering, leveraging emerging tools from synthetic biology and artificial intelligence to address the intertwined issues of cost, fidelity, standardization, and mechanistic understanding.

### 10.1 Rational metabolic engineering and hybrid architectures

**Fig. 12:**
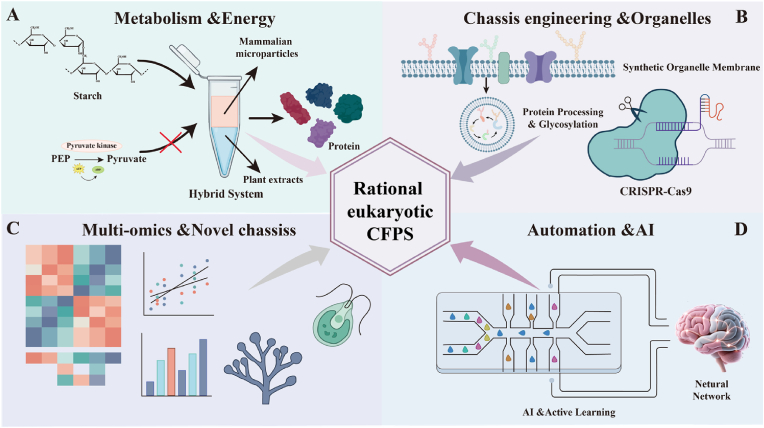
Prospects of eukaryotic CFPS system. (A) Metabolic modules incorporating low-cost carbohydrates such as starch, as opposed to relying on expensive high-energy phosphate substrates. Hybrid systems integrating performance and functionality are developed by combining translational mechanisms from plant extracts with specific synthetic pathways or microsomes within mammalian cells. (B) Engineering chassis cells using genome editing tools yields lysates capable of producing immunocompatible proteins through genetically programmed expression. Incorporating synthetic organelles or membrane vesicles to reconstruct cellular compartments enables precise embedding of glycosyltransferases and transporters within these vesicles, providing spatial isolation and an intracellular-like environment for accurate protein modification. (C) Multi-omics approaches combined with machine learning help decipher the complex nonlinear relationships between lysate composition and protein synthesis performance, yielding comprehensive datasets essential for constructing predictive models of cell-free reactions. Genome data mining identifies novel eukaryotic chassis, such as specific fungal or algal strains, expanding the range of platforms beyond existing standard systems. (D) Artificial intelligence-driven automation technology defines standardized processes for systems, thereby supporting efficient screening of protein arrays.

To overcome the trade-off between yield and economic viability, strategies such as redesigning the extract's energy metabolism module and developing hybrid systems hold promise for achieving high-quality protein output while reducing production costs (Fig. 12A). Rather than relying on costly high-energy phosphate substrates, synthetic biology offers strategies to engineer self-sustaining energy regeneration pathways. For instance, introducing orthogonal metabolic modules that utilize low-cost carbohydrates, such as starch or maltodextrin, can mimic cellular glycolysis *in vitro*, significantly reducing operational costs. The availability of computational methods for cell-free metabolic modeling will provide valuable insights into identifying metabolic bottlenecks in cell-free amplification. Furthermore, the development of hybrid systems presents a pragmatic solution to the efficiency paradox. By fusing the robust, cost-effective translation machinery of plant extracts with specific folding components or microsomes derived from mammalian cells, researchers can potentially decouple biological complexity from production costs, achieving high-quality protein output without the expense associated with mammalian cell culture.

### 10.2 Reconstructing cell compartments and chassis engineering modifications

Overcoming PTM fidelity limitations requires moving beyond simple enzyme addition. Instead, it necessitates reconstructing cellular compartments and modifying the source chassis through genetic engineering to provide the essential spatial isolation and modification environment for synthesizing high-quality proteins (Fig. 12B). The eukaryotic CFPS platform advances artificial cell design by incorporating synthetic organelles or membrane vesicles that mimic the endoplasmic reticulum and Golgi apparatus. Advances in membrane engineering allow for the precise co-embedding of glycosyltransferases and transporters within these vesicles, providing the necessary spatial isolation and non-reducing environments for correct disulfide bonding and complex glycosylation. Additionally, engineering modifications to the source chassis prior to extraction represent a reasonable solution. By using genome-editing tools to knock out plant-specific glycosylation pathways or to knock in humanized variants in the host organism, the resulting lysate can be genetically predisposed to produce immunologically compatible proteins, thereby enhancing the biomedical utility of the system.

### 10.3 Mechanistic elucidation and rational expansion of chassis diversity

To illuminate the metabolic “black box” of lysate and expand chassis diversity, it is necessary to integrate multi-omics data with machine learning to construct predictive models guiding rational energy network design, while simultaneously exploring novel eukaryotic organisms with inherent advantages to overcome limitations of standard systems (Fig. 12C). Specifically, integrating metabolomics, proteomics, and flux analysis generates comprehensive datasets for constructing cell-free reaction prediction models. Machine learning then deciphers complex nonlinear relationships between lysate composition and protein synthesis performance, enabling rational energy network design beyond trial-and-error approaches. Simultaneously, bioinformatics-guided exploration of genomic data will facilitate the selection of novel eukaryotic chassis, such as specific fungi or algae with naturally ideal traits, such as thermotolerance or high secretion capacity, thereby expanding the platform landscape beyond current standard systems.

### 10.4 Digitization and intelligent automation of workflows

Finally, the transition from laboratory processes to standardized applications requires integrating digital technologies. The inherent variability of biological extracts can be managed through the adoption of automated, high-throughput platforms driven by artificial intelligence. By combining microfluidic liquid handling with active learning algorithms, it becomes possible to screen thousands of reaction conditions with minimal sample consumption and iteratively optimize parameters in a closed loop (Fig. 12D). This data-driven approach removes human bias and operational inconsistency, facilitating the establishment of robust standard operating procedures. Such automation is particularly critical for scaling up applications in protein array fabrication and enabling the deployment of portable, freeze-dried CFPS systems for point-of-care diagnostics in resource-limited settings.

## 11 Conclusion

This paper systematically reviews and summarizes the evolutionary history, core components, and cutting-edge applications of eukaryotic cell-free protein synthesis systems. It then summarizes the fundamental components of this system, including cellular extracts with translational activity, exogenous DNA templates, and energy regeneration systems as key reaction elements. Subsequently, it thoroughly discusses and compares the characteristics and advantages/disadvantages of different eukaryotic host platforms, including extracts from protozoa, fungi, plants, insects, and mammalian cells, as well as the fully recombinant PURE system. The core of this review lies in the detailed exposition of the system's formidable capabilities in addressing critical challenges in life sciences and biotechnology. It further highlights the breakthrough applications in both fundamental research, such as genetic circuits and artificial cell construction, and applied studies, such as protein engineering and metabolic engineering, demonstrating its immense potential as an open, flexible, and controllable platform.

Despite the promising outlook, the development of eukaryotic cell-free systems remains challenging. The primary issue is the difficulty in balancing yield and cost, as protein yields are typically lower than in prokaryotic systems, and preparing extracts and reaction reagents is costly. Second, the systems exhibit limitations in precisely regulating post-translational modifications, with both modification types and fidelity still falling short of those in natural mammalian cells, particularly in the inefficient folding and assembly of membrane proteins. Furthermore, the entire field remains constrained by insufficient system standardization and automation, as well as incomplete exploration of novel host chassis cells and their underlying mechanisms.

To address these challenges, our future outlook focuses on multi-level synergistic innovation. At the fundamental level, it must deeply integrate multi-omics technologies and artificial intelligence to reveal the coupling mechanisms between the system's energy metabolism and protein synthesis, while exploring novel chassis with specialized functions. At the engineering level, efforts should focus on developing high-performance hybrid systems, designing low-cost energy regeneration solutions based on renewable resources, and achieving one-stop, high-throughput production from template to product through deep integration with microfluidic and automated platforms, thereby fundamentally overcoming current bottlenecks.

The eukaryotic CFPS system will transcend its traditional role as a laboratory research tool to become a core engine driving fundamental scientific discoveries and industrial application innovations. In fundamental science, it will provide an unprecedented “test-tube” research platform for deciphering the structure and function of complex proteins, simulating intracellular environments, constructing minimal artificial cells, and exploring the origins of life, thereby deepening our understanding of the principles governing life processes. At the industrial application level, this system will significantly empower the biopharmaceutical industry, enabling rapid vaccine production for emerging infectious diseases, on-demand manufacturing of personalized therapeutic antibodies, and the development of portable diagnostic devices. Simultaneously, it will play a pivotal role in green biomanufacturing by enabling the synthesis of high-performance enzyme catalysts and biomaterials. Through continuous technological iteration and interdisciplinary integration, the eukaryotic cell-free system is poised to usher in a new era of efficiency, precision, and customization for synthetic biology and biomanufacturing.

## CRediT authorship contribution statement

**Shengxin Tong:** Writing – original draft, Methodology, Investigation, Formal analysis. **Yiming Wang:** Methodology, Investigation. **Xin Wang:** Methodology, Investigation. **Xiaowen Jin:** Methodology, Investigation. **Duoduo Tan:** Methodology, Investigation. **Ze Wang:** Supervision, Project administration. **Yuan Lu:** Writing – review & editing, Supervision, Project administration, Funding acquisition, Conceptualization.

## Declaration of competing interest

The author Yuan Lu is an Editorial Board Member for Synthetic and Systems Biotechnology and was not involved in the editorial review or the decision to publish this article. Other authors declare that they have no known competing financial interests or personal relationships that could have appeared to influence the work reported in this paper.

## Data Availability

All data supporting the conclusions of this study are derived from the cited literature, and no new datasets were generated.
